# Feasibility of a home-based home videogaming intervention with a family-centered approach for children with cerebral palsy: a randomized multiple baseline single-case experimental design

**DOI:** 10.1186/s12984-024-01446-2

**Published:** 2024-09-04

**Authors:** Daniela Chan-Víquez, Heilyn Fernández-Huertas, Carles Montserrat-Gonzalez, Ajmal Khan, Darcy Fehlings, Sarah Munce, F. Virginia Wright, Elaine Biddiss

**Affiliations:** 1 Bloorview Research Institute, Holland Bloorview Kids Rehabilitation Hospital, Toronto, Canada; 2 Rehabilitation Sciences Institute, University of Toronto, Toronto, Canada; 3 Escuela de Tecnologías en Salud, Universidad de Costa Rica, San José, Costa Rica; 4 Department of Paediatrics, University of Toronto, Toronto, Canada; 5 KITE-Toronto Rehabilitation Institute, University Health Network, Toronto, Canada; 6 Institute of Biomedical Engineering, University of Toronto, Toronto, Canada

**Keywords:** Cerebral palsy, Exergaming, Family centred approach, Telerehabilitation, Developing countries, Health services accessibility, Single-case studies

## Abstract

**Background:**

Worldwide, children with cerebral palsy (CP) living in underserved communities face barriers to accessing motor therapy services. This study assessed the implementation and effectiveness of an 8-week, upper limb (UL) home-based intervention with a movement-tracking videogame (Bootle Blast) in Costa Rican children with CP.

**Methods:**

Children established a weekly playtime goal and two UL activities of daily living (ADLs) that they would like to improve on. A multiple-baseline, single-case experimental design, was used with the Performance Quality Rating Scale (PQRS) as the repeated measure to track changes in performance of the selected ADLs between the baseline (usual care) and intervention (Bootle Blast) phases. The Canadian Occupational Performance Measure (COPM), the Box and Blocks Test (BBT) and the Children’s Hand-Use Experience Questionnaire (CHEQ) were collected before and after the intervention. Technical barriers were documented during weekly video calls with a monitoring therapist. Treatment effect size, slope changes and percentage of non-overlapping data were identified for the PQRS. Descriptive statistics summarized results for the BBT, CHEQ, videogame logs (e.g., playtime) and technical barriers.

**Results:**

Fifteen children participated and 13 completed the intervention. Both participants who dropped out did so after completing baseline assessments, but before experiencing Bootle Blast. Children’s mean *active* playtime (i.e., mini-games targeting the UL) across the 8-weeks was 377 min, while mean *total* time spent engaging with Bootle Blast (*active* + *passive* play time [e.g., time navigating menus, reviewing rewards]) was 728 min. In total, eight technical issues (from five children) were reported, and all but three were resolved within 48 h. Partial effectiveness was associated with the intervention. Specifically, 85% of participants improved on the PQRS and 69% achieved clinically important improvements ≥ 2 points in performance on the COPM. Children improved by 1.8 blocks on average on the BBT, while on the CHEQ, five children had a clinically important increase of 10% of the total number of UL activities performed with both hands.

**Conclusion:**

Bootle Blast is a feasible and effective option to facilitate access and engage children with cerebral palsy in UL home rehabilitation.

*Trial registration* Trial registration number: NCT05403567.

**Supplementary Information:**

The online version contains supplementary material available at 10.1186/s12984-024-01446-2.

## Introduction

Cerebral palsy (CP) is the most common neuromotor childhood disability affecting 1.6 (high-income countries) to 3.4 (low- and middle-income countries [LMICs]) in 1000 children [1]. It is a movement and posture non-progressive disorder impacting motor skills performance, and the ability to carry out daily life activities (ADLs) independently [2]. Rehabilitation motor therapies can improve quality of movement and participation in ADLs, facilitating social inclusion in children with CP [3]. Yet, worldwide, children with CP living in low-income families, rural areas and developing countries face barriers in accessing motor therapy services [4, 5].

In Costa Rica, over half a million children live with a disability [6, 7], and 43% of these experience difficulties accessing healthcare services [7, 8]. In our previous research with Costa Rican children with CP we learned that only six of 15 participants had ever received rehabilitation for the upper limb (UL). Economical constraints such as not being able to afford UL therapy, or having to prioritize other health services were the primary accessibility barriers reported, followed by time constraints (e.g., difficulty fitting conventional therapy into the child’s schedule), geographical (e.g., need to travel long distances to access care) and COVID-related barriers (e.g., limited availability of services) [9].

Movement-tracking videogame interventions that engage children in UL therapy at home, may help bridge these accessibility gaps. Some videogaming technologies can be integrated in telerehabilitation programs that allow for remote monitoring and adjustment of different game parameters (e.g., level of difficulty, range of movement) [10]. Videogaming interventions can also provide new avenues to engage and motivate children in doing therapy, as they are usually perceived as fun [11, 12] and can ease parental burden as caregivers do not have to be trained to deliver the intervention or be the “therapist at home” [13]. Moreover, they have shown positive results regarding improvements in UL motor functional outcomes and quality of life when used for home-based rehabilitation in children with CP [14–16].

Bootle Blast is a videogame comprised of 13 mini-games targeting motor skill development of the UL (e.g., reaching, grasping-releasing, wrist supination-pronation). Movements are tracked via a 3D camera-computer (Orbbec Persee®). Some of the mini-games are “mixed reality” wherein real-life objects are manipulated (e.g., building blocks, musical instruments) to play the game. Bootle Blast does not require internet connection and can be easily connected to a standard TV screen or monitor. Bootle Blast’s automated set up involves a calibration game, where the range of movement for each shoulder, elbow, and hand is determined. During this process, the targeted UL that will be used in unilateral games is identified, while therapeutic objectives and a weekly *active playtime goal* (i.e., time the child spends actively engaging in therapeutic movements during mini-games [aPTG]) are set by the study’s monitoring therapist alongside the parent and child [17]. The videogame also records *passive playtime*, which includes the time spent engaging with Bootle Blast outside of the mini games (e.g., navigating menus, reviewing rewards). While *active playtime* intensely targets specific therapeutic movements, *passive playtime* also involves some movement and motor control to navigate menus and make selections.

In Bootle Blast, short (e.g. score counts), mid- (e.g. unlocking new game content) and long (e.g. collecting 100 “rare bootles” to finish the game) term rewards are designed to promote player engagement and are linked to the individual’s abilities and aPTGs as described in detail in previous work [17]. Pilot work with Bootle Blast when used with children with CP in Canada, has provided a firsthand understanding of how this videogame can be integrated into home use and what supports are needed [17]. However, while North American and European literature largely support the use of movement-tracking videogames for home rehabilitation in pediatric populations [18], the feasibility of these interventions in developing countries still needs to be explored.

This paper reports on a sub-set of results associated with an overarching feasibility, multi-phase mixed methods project (NCT05403567) (Appendix 1). Specifically, the objectives of this study were (1) to evaluate *implementation* (i.e., the extent to which it is possible for an intervention to be implemented as planned) [19] via the amount of time the children spent playing Bootle Blast and the number of reported technical barriers, and (2) to determine the extent to which the 8-week home-based intervention with Bootle Blast was *effective* in improving UL functional outcomes in Costa Rican children with CP.

## Methods

### Study design

This study used a feasibility design [19] to evaluate *implementation*, alongside a single case experimental design to establish *effectiveness*. Integrating a time series design expands beyond the traditional pre-post approaches of feasibility studies, allowing for the establishment of outcome indicators, and providing a true measure of observed changes. Specifically, we used a randomized multiple-baseline, single case experimental design in which a randomized baseline phase (phase A, 3–5 weeks) was followed by the Bootle Blast intervention (phase B, 8 weeks) with additional pre and post assessments of hand function. Using this approach, a trend of behavior was established in the baseline phase prior to starting the intervention phase in order to mitigate threats to internal validity [20]. The randomization was performed with the *randbetween* function in Microsoft Excel. Of note, this study was designed and partially conducted during the Coronavirus pandemic, and as such, the intervention and clinical research assessments were selected to support remote administration. The Template for the Intervention Description and Replication—telehealth [21] and the Single-Case Reporting Guideline in Behavioural Interventions (SCRIBE) [22] checklists were followed in this manuscript.

### Participants and sampling

During phase 1 of the overarching research study [9], Costa Rican children with CP were invited to participate alongside a parent. A voluntary, convenience sample (n = 15) was recruited via social media platforms (i.e., Instagram, Facebook, WhatsApp) and word of mouth across the country. Inclusion criteria for phase 1 were:

- Diagnosis of CP, 7 to 17 years of age—the minimum age was chosen based on previous experiences with Bootle Blast. While it varies by individual, children aged 7 years and up are typically able to understand how to play the game and have sufficient height to be tracked accurately by the Orbbec Persee camera.
- Manual Ability Classification System (MACS) levels I (objects are handled easily and successfully), II (handles most objects but with some reduced quality and/or speed), and III (handles objects with difficulty—the child will need help to prepare and/or modify activities) [23]. The MACS was assessed via videocall by a clinician-researcher (DC) and determined based on parent report.
- Caregiver willing to participate (i.e., assist during virtual clinical assessments, participate in interviews).
- Able to communicate verbally in Spanish or English.
- Access to a TV screen or monitor at home.
- Ability to cooperate, understand, and follow simple instructions for game play as reported by the parent.
- Has an accessibility barrier to UL rehabilitation services as reported by the parent (e.g., not able to pay for therapy, services not available in their area).

Children were excluded if they had a history of uncontrolled epilepsy, visual or hearing impairments that limited their ability to play Bootle Blast, had received constraint induced movement therapy or botulinum toxin injections in the past six months, or active therapy of the UL within three months of the study enrollment.

For this study (phase 2), a nested sample [17] from phase 1 [9] was used. Additional eligibility criteria to participate in phase 2 were:

- The family expectations and the child’s therapy goals were in line with the scope of the Bootle Blast intervention.
- The child-parent dyad was able to commit to an aPTG of at least 45 min a week [17] as established during the phase 1 interview [9].
- The child could successfully play at least 10 of the 13 mini-games (assessed during the onboarding session, see *data collection: protocol*).

In feasibility studies, small convenience samples are used to estimate effect sizes, power, and sample sizes for future larger trials [19]. A randomized clinical trial is currently underway to assess the effectives of Bootle Blast in children with motor disabilities in Canada. In single case experimental designs, a minimum sample of three to five participants is required [20]. As this study combines both intervention designs, a sample of 12–15 participants was considered sufficient to assess the feasibility of implementation and effectiveness of the Bootle Blast intervention.

### Data collection

#### Implementation (feasibility) indicators

Throughout the 8-week intervention phase, the Bootle Blast system’s game logs recorded details for each play session including *active* (i.e., minutes spent in the mini-games) and *passive* (e.g., time navigating menus) playtimes, game scores, games played, and system events to aid in identifying and resolving technical issues (e.g., videogame not loading). When the system was periodically connected to the internet, these logs were automatically uploaded onto the cloud and accessible remotely to the researchers. Technical assistance requests reported to a monitoring therapist (DC) during weekly video calls with the child and the parent (see *protocol*) were used as a secondary indicator to address the feasibility of *implementation*.

#### Effectiveness outcome measures

The Performance Quality Rating Scale (PQRS) [24] and the Canadian Occupational Performance Measure (COPM) [25] were the co-primary outcome measures used to evaluate *effectiveness*. Secondary measures of UL use were the Box and Blocks Test (BBT) [26] and the Children’s Hand-Use Experience Questionnaire (CHEQ) [27]. All assessments had official translations in Spanish, and these versions were administered to participants by a Costa Rican physiotherapist (DC).

The PQRS is a clinician-rated, observational scale that evaluates performance on client-selected, video-recorded ADLs. The PQRS General Scale uses a 10-point response scale (1 = “can’t do the skill at all”, 10 = “does the skill very well”) for timeliness of completion and quality of performance. Scores from these two domains are then averaged to identify the overall quality of activity performance. It has been used with children with diverse diagnoses, with excellent test–retest reliability (> 0.9) across time periods and multiple raters, with an average smallest real difference of 2.55 points. Internal responsiveness is high with large effect sizes reported [24]. The PQRS served as the repeated measure for the effectiveness outcomes. This measure was scored based on weekly videos of the child performing two meaningful ADLs involving the UL that could be done at home. These ADL goals were identified as part of an interview conducted in Phase 1 of the overarching study [9]. In summary, goals were established through a collaborative conversation with the main stipulation being that they must be filmable in the home setting.

The COPM is a patient-reported measure that evaluates performance and satisfaction with performance on ADLs identified by the child and/or parent to be meaningful. The parent rated pre and post intervention the child’s level of performance and satisfaction on a 10-point scale (1 is low, 10 is high) for each of the two ADL goals identified in the phase 1 interviews [9]. The COPM has shown good reliability, construct validity and responsiveness (minimally clinical importance difference [MCID] of two points) when used with children with CP [25]. Of note, the PQRS and COPM can complement each other when they are used to evaluate the same activities, which was the case in this study. The PQRS provides information on what the child can do in a test context, while the COPM rated by the parent, reflects the child’s performance of that activity in daily life.

The BBT [26] and the CHEQ [27] were secondary outcome measures administered pre and post intervention. The BBT consists of 150 wooden cubes—2.5 cm in size within a wooden box that has two open compartments with a vertical divider separating them. Unilateral gross manual dexterity is measured by having the participant pass as many cubes as possible above the division, from one side to another, in 60 s. The test is appropriate for ages three and up. The BBT has excellent test–retest reliability (ICC ≥ 0.85) and moderate responsiveness (effect size ≥ 0.75, more affected hand) in children with CP [28].

The CHEQ captures perceived quality and effectiveness of the child’s use of their affected hand in bimanual task performance in 27 ADLs [27]. In this study, the CHEQ was completed by the parent. It is scored in a unit scale from 0 to 100 with higher scores reflecting better abilities. Additionally, it provides a count of the number of activities the child can perform bimanually, with one hand or with help. The CHEQ has been reported to be valid and reliable (test–retest, ICC 0.87–0.91) for children with CP [29], with a 10% increase for the “*activities performed with both hands*” component indicating a clinically important change [30].

#### Protocol

##### Baseline (phase A)

UL ADL videos for the PQRS were recorded weekly over the participant’s 3 to 5 week baseline phase (as randomized) during a video call (Zoom, same day each week). In the first video call, the families were instructed on how to record the videos independently if needed; if there was a week(s) when a video call was not possible, the parent recorded the videos and sent them via WhatsApp to the monitoring therapist. Only videos sent within a ± 2-day window from their usual video call day were included in the analysis.

Prior to the final week of the baseline phase, a Bootle Blast welcome package was delivered to the participants’ homes. This package included: (1) the Orbbec Persee with Bootle Blast installed, (2) a user manual with explanations and troubleshooting tips, (3) toy musical instruments (i.e., tambourine, castanet, maraca, and glockenspiel) and coloured building blocks (Mega Blocks) to play the mixed-reality games, and (4) a BBT set constructed from cardboard and blocks with dimensions as specified [26]. Additionally, a questionnaire was completed by the parent to document demographic characteristics.

In the final week of the baseline phase, the BBT was administered by DC (monitoring therapist) via video call, and parents completed the CHEQ and the COPM via REDCap [31]. Initial instructions on how to fill out the measure (i.e., a practice one-item trial as suggested by the CHEQ guideline) were provided by DC for the CHEQ. For the COPM, DC and the parent first went through an example together (different from the child’s chosen ADLs) on how to complete it, and then, the parent rated performance and satisfaction for the two ADLs selected as the child’s COPM goals. Of note, the same ADLs were also used for the PQRS videos. The onboarding session with Bootle Blast also took place in this video call, during which DC gave instructions on how to calibrate, use and play the game at home. DC then guided the child on how to input their desired weekly aPTG into the system.

For the observational measures, the BBT was scored by DC from the Zoom video. She was blinded at post scoring (not able to access pre-intervention scores, post assessment video was scored six months after the pre-assessment). The PQRS was scored by a trained physiotherapist (CM) blinded to the timepoint at which the test was administered. To ensure blinding, videos were edited to delete the date on which they were taken, or any verbal cue that could suggest it. The assessor had access to all the videos (baseline, intervention, and post) and completed the scoring for each child within three days.

##### Home intervention (phase B)

Children played Bootle Blast at home for 8 continuous weeks. Despite having a weekly aPTG, parent and child (participant dyad) were reminded that children could play as much as they could or wanted. At the beginning of each gameplay session, a randomized playlist of three mini-games (three minutes each) was displayed on the screen to encourage participants to try the full range of games available. After completing the playlist, children had complete autonomy over which mini-games they played.

The monitoring therapist, DC, scheduled weekly video calls (approximately 15 min) with each dyad to check-in, record the UL ADLs videos for the PQRS, answer questions, troubleshoot any technical problems, and identify possible factors influencing engagement in Bootle Blast play. The content of each of these interactions, technical assistance requests, and the therapist’s views on the challenges/benefits faced by the participants were documented in field notes.

At the end of week 8, the videogame locked to prevent further play until the post-intervention assessments were complete which consisted of the same battery of tests (i.e. COPM, CHEQ, BBT and PQRS). Table 1 depicts the time points at which assessments were administered. Of note, after completing the research study, children were able to keep Bootle Blast and the Orbbec Persee for personal use.

**Table 1:** Sample participant involvement timeline and time points at which assessments were administered (3–5 weeks baseline)

Example 1	Baseline (phase A, 5 weeks)					Intervention	Post
Week	1	2	3	4	5	6–13	14
PQRS	x	x	x	x	x	x	x
COPM, BBT, CHEQ,					x		x
Onboarding					x		
Demographic questionnaire				x			

Example 2	Baseline (phase A, 4 weeks)					Intervention	Post
Week	1	2	3	4	–	5–12	13
PQRS	x	x	x	x	–	x	x
COPM, BBT, CHEQ,				x	–		x
Onboarding				x	–		
Demographic questionnaire			x		–		

Example 3	Baseline (phase A, 3 weeks)					Intervention	Post
Week	1	2	3	–	–	4–11	12
PQRS	x	x	x	–	–	x	x
COPM, BBT, CHEQ,			x	–	–		x
Onboarding			x	–	–		
Demographic questionnaire		x		–	–		

*PQRS* Performance Quality Rating Scale, *COPM* Canadian Occupational Performance Measure, *BBT* Box and Blocks Test, *CHEQ* Childrens’ Hand-Use Experience Questionnaire, x symbol indicates timepoints at which the assessment was administered, – symbol indicates an empty cell (i.e., no baseline week)

### Data analysis

Success criteria to evaluate the feasibility of *implementation* and the *effectiveness* of the Bootle Blast intervention were developed a priori with reference to previous studies of similar UL home-based interventions in children with CP [17, 32, 33].

Success criteria for the main feasibility of *implementation* indicators were as follows:

- ≥ 80% of children would complete the intervention.
- ≥ 80% of children who completed the intervention would achieve their weekly aPTG in ≥ 6 weeks.
- ≤ 20% of participants would experience technical barriers preventing them from playing ≥ 4 days.

Videogame logs were reviewed, outliers (e.g. instances where the game had accidentally been left on and running) were removed and descriptive statistics for *active* and *total* (i.e., *active* + *passive*) playtime were calculated. Game logs were also used to determine the percentage of children who met their weekly aPTG, and on how many weeks this goal was achieved. To understand the nature and frequency of technical issues encountered, the monitoring therapist’s notes were analyzed using content analysis [34] and reported alongside the videogame log data.

Success criteria for the clinical outcome measures (*effectiveness*) were as follows:

- ≥ 75% of the children who completed the intervention would show a positive change on at least *one* of their identified UL ADLs:
  - For the PQRS, a small (0.2–0.5) to moderate (0.5–0.8) effect size [35] would reflect a positive change.
  - For the COPM, a positive change was a pre-post increase of 2-points (MCID) [25] in the perceived performance.

PQRS individual improvement was calculated as: highest score in phase B—highest score in phase A [24]. Wilcoxon-signed rank was used for descriptive statistics of overall improvement and effect size (rB). Individual effect sizes (standard mean difference [SMD]) were given by: (mean of phase B − mean of phase A)/SD of phase A [24]. Slope changes were visually identified using the split-middle trend line and mean level [20]. The degree of association between time points during the baseline phase was checked using the Tau-U method, and the baseline trend was corrected if needed [36, 37] followed by identifying the percentage of non-overlapping data (PND) [38].

Pre-post differences on the COPM scores were established using a paired sample t-test (SMD and 95% CIs). The BBT was interpreted based on an increase of two blocks on the more affected hand at post [39] and the CHEQ based on a 10% score increase for the “activities performed with both hands” component [30]. Statistical analyses were conducted using JASP 0.17.1 software.

## Results

### Participants

**Fig. 1:**
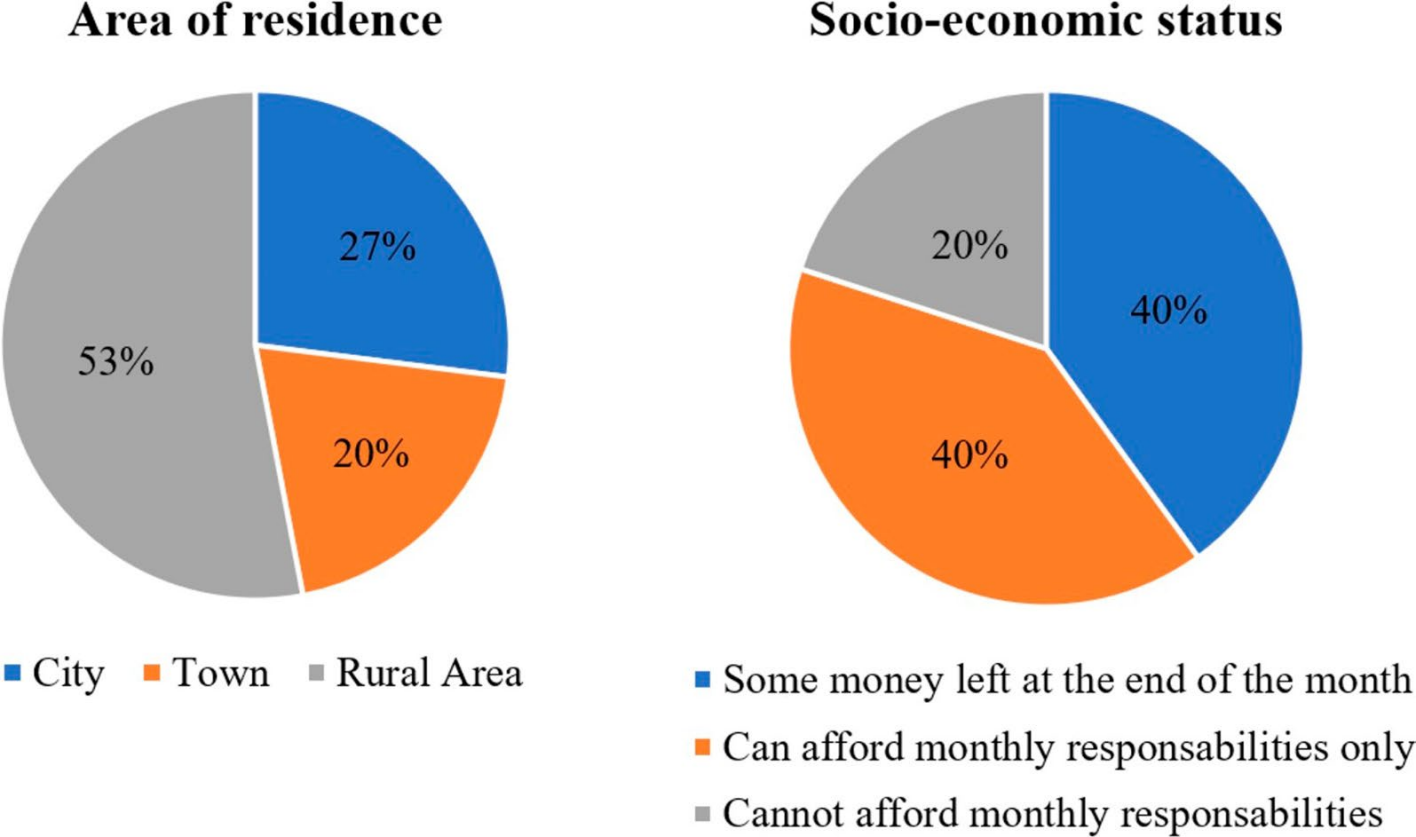
Participants’ (%) area of residence and socio-economic status (n = 15)

Fifteen children (10.3 ± 2.6 years) from across Costa Rica and one of their parents participated in the study as described in Table 2 and Fig. 1. Nine child participants had sibling(s) living in the same household, most of them being young or school-aged children. Two children (participants 3 and 4) withdrew at the end of the baseline phase, after completing all clinical assessments but before experiencing Bootle Blast. Reasons for leaving the study are unknown for participant 3 as contact was lost with the family. For participant 4, a family situation delaying the start of phase B resulted in the dyad losing interest in continuing.

**Table 2:** Participants’ demographic characteristics

*ID*	Sex	Age (years)	Participating parent	MACS level	Most affected side	Diagnosis	Videogame experience^a^
*1*	M	13	Mother	III	Left	Quadriplegia*	No
*2*	M	8	Mother	II	Right	Hemiplegia*	Plays sometimes
*3*	F	8	Mother	III	Right	Triplegia*	Plays sometimes
*4*	M	11	Mother	III	Right	Triplegia*	Plays sometimes
*5*	M	7	Mother	II	Left	Hemiplegia**	Plays every week
*6*	F	8	Mother	I	Left	Hemiplegia*	Plays sometimes
*7*	F	7	Father	III	Right	Quadriplegia*	No
*8*	M	10	Mother	I	Left	Hemiplegia*	Plays sometimes
*9*	M	9	Mother	II	Left	Hemiplegia**	Plays sometimes
*10*	M	13	Mother	III	Left	Hemiplegia**	Plays every week
*11*	M	10	Mother	I	Left	Hemiplegia*	Plays every week
*12*	M	12	Mother	II	Left	Triplegia**	Plays every week
*13*	M	10	Mother	II	Right	Hemiplegia*	Plays every week
*14*	F	16	Father	III	Left	Quadriplegia*	Plays sometimes
*15*	F	12	Mother	II	Right	Triplegia*	Plays sometimes

^a^Movement or hand-held controller. M, male; F, female; MACS, Manual Ability Classification System. *as reported by the parent or **by a therapist who had treated the child in the past

### Feasibility of implementation

#### Playtime goal

**Fig. 2:**
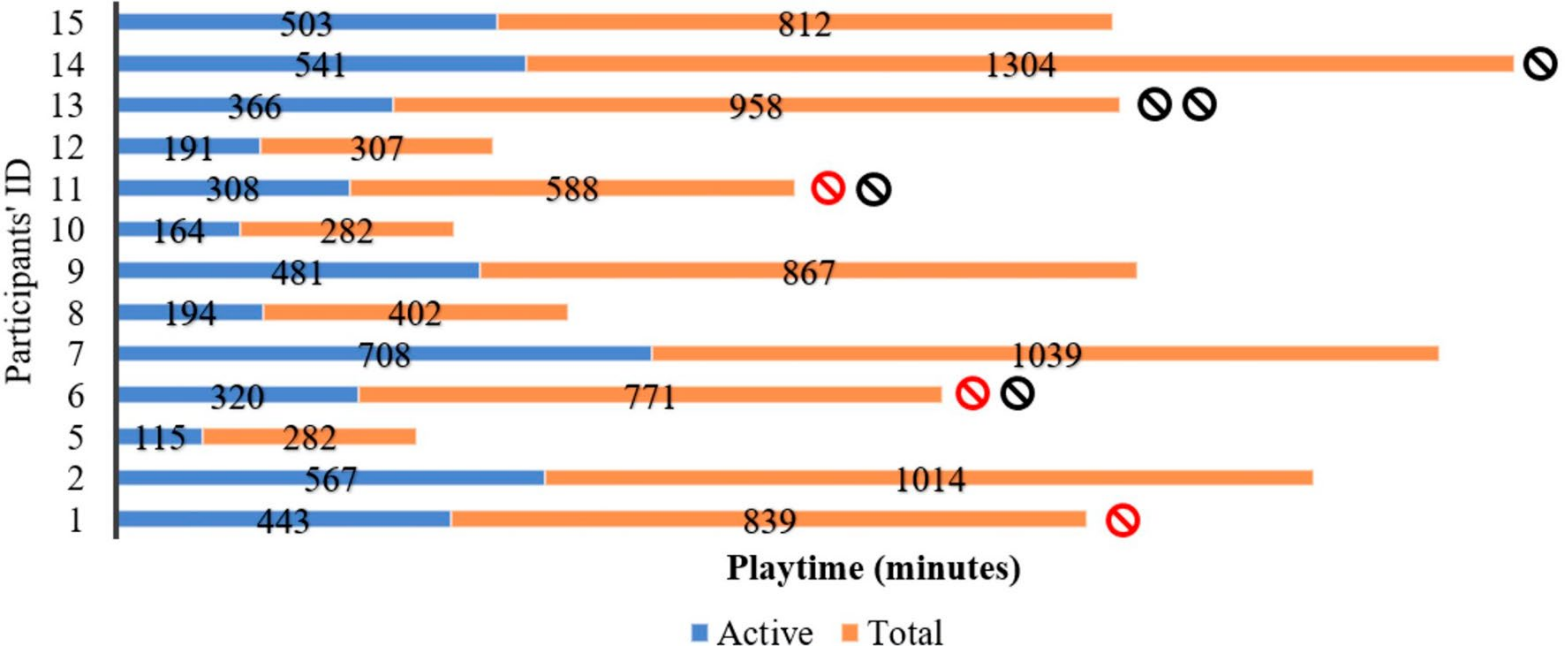
Participants’ individual playtimes throughout the intervention (n = 13). Red symbol represents participants with technical issues preventing them from playing ≥ 4 days at some point in the 8 week play cycle. Black symbol represents technical barriers solved within 48 h

Following the onboarding session with Bootle Blast, dyads identified a mean weekly aPTG of 45–80 min (60 ± 15). Success indicators were partially met as 87% (13/15) of children completed the intervention, but only 23% (3/13) achieved their aPTG ≥ 6 weeks. Children played on average 16 ± 6 days (twice a week) across the 8-weeks for a total of 377 ± 181 min of *active* playtime (23 ± 6 min per session) and 728 ± 330 of *total* playtime (*active* [playing mini games] + *passive* [e.g., navigating menus] playtime) with an average of 45 ± 11 min per session (Fig. 2). Children with quadriplegia accumulated the highest *total* intervention playtime (1061 ± 233 min) followed by children with hemiplegia (646 ± 299 min) and triplegia (560 ± 357 min). Appendix 2 shows individual playtimes by intervention week.

**Fig. 3:**
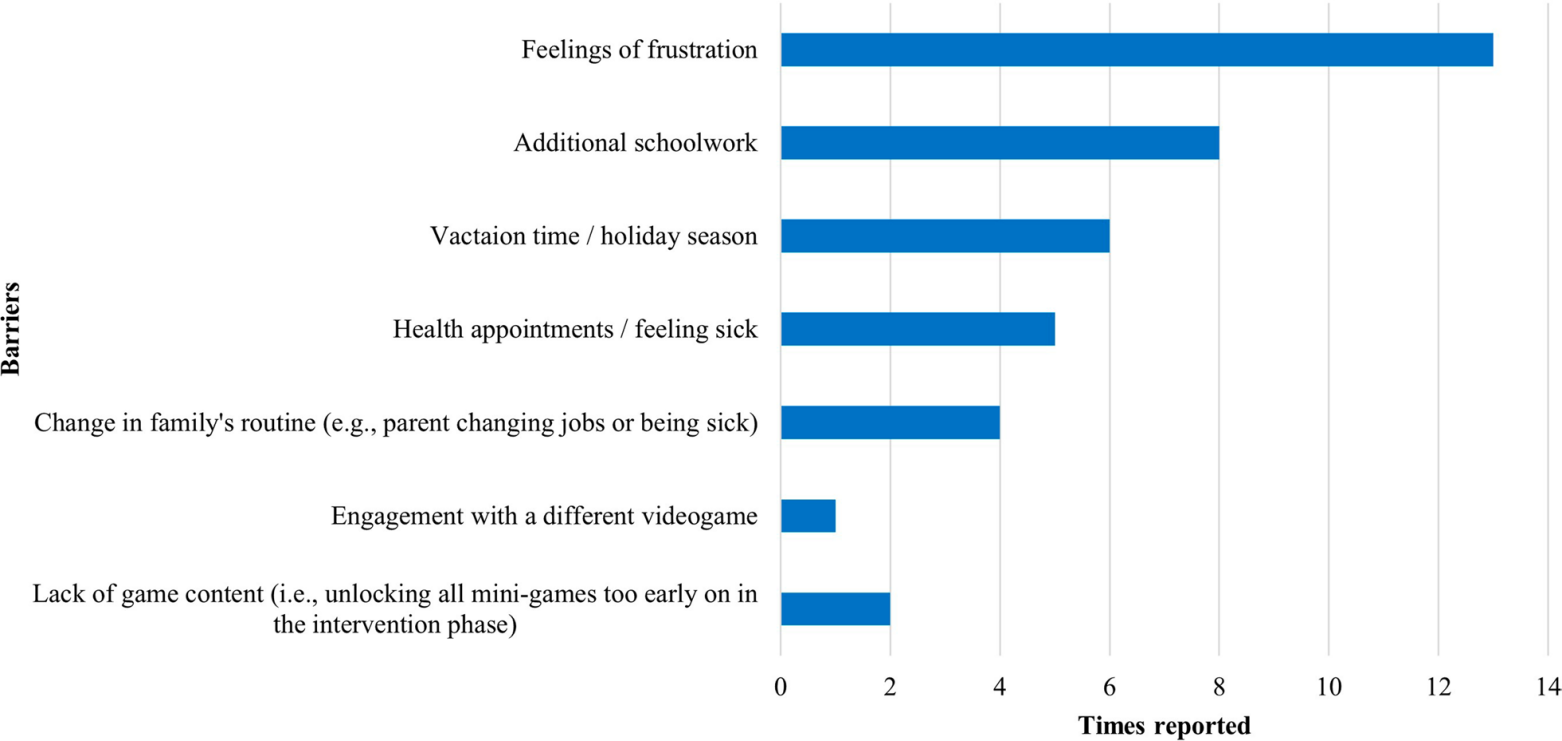
Reported barriers to engage in playtime with Bootle Blast. Times reported during 86 weekly video calls (out of a maximum of 91). Some dyads reported more than one issue during a video call, while others reported the same issue in more than one call

The monitoring therapists’ field notes made during the weekly video calls highlighted the frequency of barriers to engaging in play with Bootle Blast as reported by families (Fig. 3). The most common barrier was experiencing frustration when not understanding how to play a mini-game, or not being able to play it due to a physical or technical limitation. Additionally, the ability to choose when and how much to play and having weekly follow ups were important facilitators to motivating play. While some children reported enjoying playing with someone, some parents mentioned the multi-player option would evoke feelings of frustration in their child when they were not able to win. This was especially true for children who played with siblings.

#### Technical issues

Three children (23%) were prevented from playing ≥ 4 days due to a technical barrier, and this was slightly above the target incidence criterion of < 20% (Table 3).

**Table 3:** Detail on reported technical problems across participants (n = 5) throughout the intervention

Technical issue	# of times reported	Time to resolve	Modality to resolve	Participant prevented from playing ≥ 4 days
Game freezing or “stuck” (i.e. video is frozen and requires reset)	5	within 48 h (n = 2)	Remotely	No
		5 days (n = 2)	Remotely (n = 1) In person (n = 1)	Yes
		8 days (n = 1)	Remotely	Yes
Mini games not unlocking after achieving the required level	2	within 48 h	Remotely	No
System not identifying interactions with one of the mixed reality items (i.e. the maraca) correctly	1	Not resolved	Remote solution not available. By the time a software update for this issue would be ready, the participant would have finished the intervention	No, but prevented from playing Bootle Band (Appendix 3)

Technical issues related to the game freezing or being “stuck” were largely caused when Bootle Blast was left on for extended periods of time, as some participants (n = 3) would only turn off their TVs instead of exiting the game and turning off the Orbbec system. As a resolution, the researcher (DC or HF) or parent, (when addressed remotely via a Zoom call) manually extracted the files to reset progress in the game and taught the dyad how to correctly turn off the videogame.

### Effectiveness

#### PQRS

**Fig. 4:**
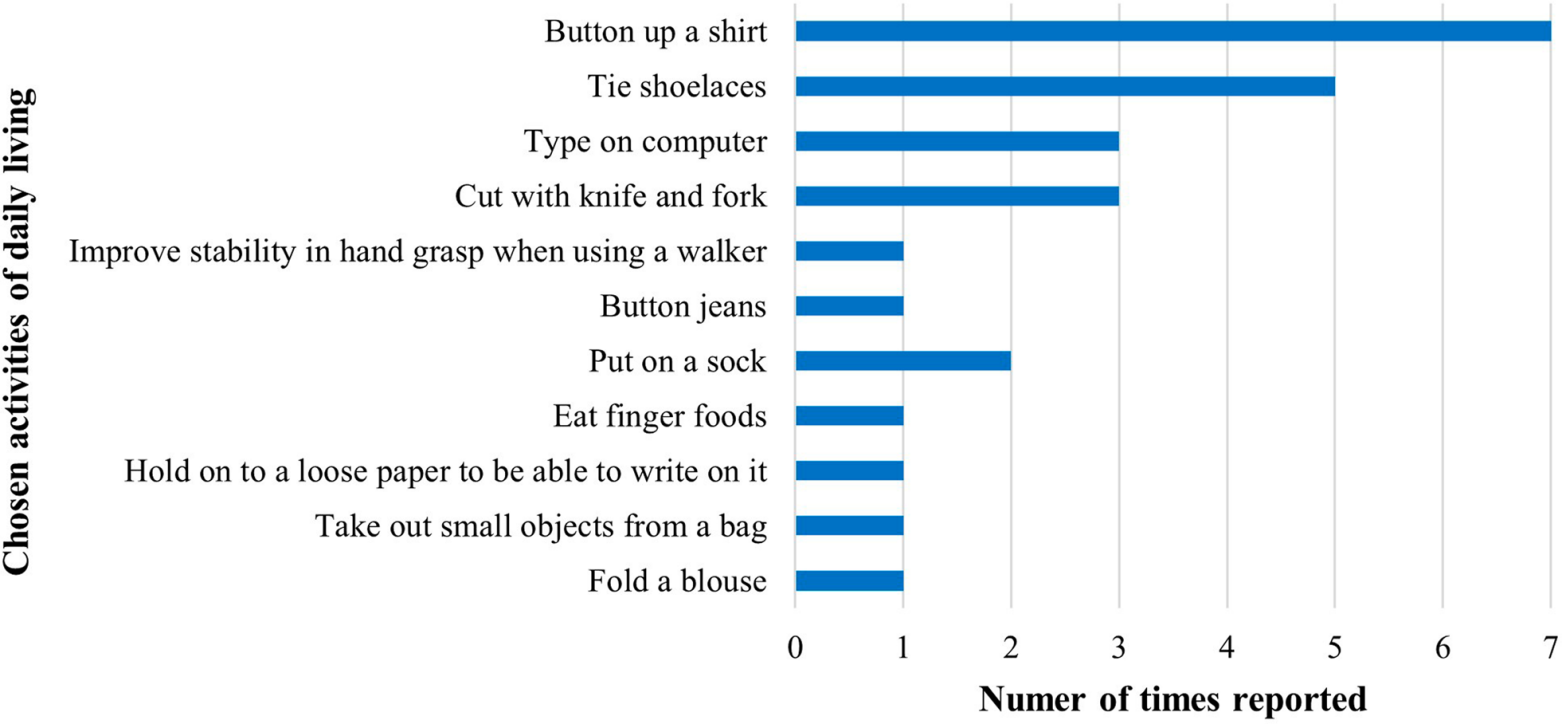
Activities of daily living (ADLs) in which participants wished to improve their UL motor function

Eleven (85%) children improved on at least one chosen ADL in the PQRS (small to high effect size), which exceeded the study success criterion of 75% (Table 4, Appendix 4). The median PQRS improvement was 1.00 ± 2.26 pts (rB = 1; p = 0.01) for ADL 1, and 0.50 ± 1.07 pts (rB = 1; p = 0.008,) for ADL 2. When the PQRS score for the ADL with the best improvement was considered, the median increase was 1.00 ± 2.17 pts (rB = 1; p = 0.004). The order of the ADLs was not necessarily reflective of the importance it held for the family. Figure 4 details the frequency of UL chosen ADLs across participating dyads.

**Table 4:** Visual analysis and descriptive statistics for the UL ADL with better PQRS improvement by participant (n = 13)

ID	Improvement (pts)	SMD	Trendline	Level change	PND (%)
1	1.5	0.83	Increasing	Higher	37.5
2	*0*	0.26	*Decreasing*	*No change*	*0.0*
5	2	0.69	Increasing	Higher	14.3
6	1	0.48	Increasing	Higher	16.7
7	0.5	− *0.65*	Increasing	*Lower*	14.3
8	0.5	*0.05*	Increasing	*No change*	16.67
9	1	–	Increasing	Higher	75*
10	8	–	Increasing	Higher	100*
11	1.5	1.00	*Decreasing*	Higher	50.0
12	4	2.08	Increasing	Higher	71.43*
13	*0*	0.33	Increasing	*No change*	*0.0*
14	0.5	0.87	*Flat*	Higher	12.5
15	1.5	1.06	Increasing	Higher	50.0

*SMD* standard mean difference, *PND* percentage of non-overlapping data; – unable to calculate as the standard deviation of the mean in phase A was 0 (i.e., data suggests a high SMD); * significant *p* value, text in italics represent negative values and/or no effect in the intervention phase

As per the SCRIBE checklist [22], inter- and intra-rater reliability were calculated using baseline videos at three time points (T1 = first baseline video, T2 = second last video before intervention, T3 = last video before intervention, n = 15). For this reliability work, a second rater blinded to the date of the video, scored the PQRS baselines. For ADL 1, inter-rater reliability was moderate to good (ICCs = 0.66 to 0.81) across all time points and for ADL 2, inter-rater reliability was poor for T1 (ICC = 0.31), and good for T2 and T3 (ICCs ≥ 0.82). Intra-rater reliability (T1, n = 14) was good for ADL 1 (ICC = 0.84) and excellent for ADL 2 (ICC = 0.97).

### COPM

Nine children (69%) achieved the COPM’s MCID of over 2 points in performance (below the success criterion of 75%) and 12 children (75%) exceeded a 2-point increase on the satisfaction scale, in at least one ADL. Overall, mean post intervention performance and satisfaction scores increased beyond the MCID of 2 points with large effect sizes for both chosen ADLs (Table 5).

**Table 5:** COPM pre-post scores (n = 13)

	Pre (mean ± SD) (/10)	Post (mean ± SD) (/10)	*p*	Cohen’s d (95% CI)
ADL 1 performance	4.54 ± 1.85	7.85 ± 1.63	< 0.001*	1.36 (0.58, 2.11)
ADL 1 satisfaction	5.77 ± 2.59	8.62 ± 1.94	0.015*	0.79(0.15, 1.40)
ADL 2 performance	5.62 ± 2.40	7.77 ± 1.36	0.011*	0.84, (0.19, 1.46)
ADL 2 satisfaction	5.62 ± 2.60	8.23 ± 1.42	< 0.001*	1.18 (0.45, 1.88)

^*^ Significant *p* value (*p* ≤ 0.05)

### BBT

Mean improvement on the BBT at the post-assessment was 1.8 ± 5.2 blocks (Appendix 5), with seven of 12 children improving by at least 3 blocks. There were missing post-test data for one child (participant 13) who was not able to complete the physical assessments due to illness. However, the parent was able to complete the self-report measures. Inter-rater (ICC 0.98, 95% CI 0.93–0.99) and intra-rater (ICC 0.99, 95% CI 0.988–0.999) reliability were excellent for the virtual assessment of the BBT.

### CHEQ

Eight of 12 children improved on the number of activities performed with both hands, with five having a positive change of ≥ 10% (Table 6). One parent did not complete the CHEQ at the post assessment (participant 8).

**Table 6:** CHEQ pre-post scores (n = 12)

	Pre (mean ± SD) (/100)	Post (mean ± SD) (/100)	*p*	Cohen’s d/Rank Biserial Correlation (95% CI)
How do you think the child's hand works?	48.0 ± 11.1	55.0 ± 8.1	0.013*	0.85, (0.17, 1.5)
How much time does your child need to do the whole task, compared to peers?	45.7 ± 10.9	47.4 ± 17.3	0.563	0.21, (0.42, 0.71)
Is your child bothered by his reduced hand/arm function during this activity	59.2 ± 12.4	76.2 ± 21.2	0.009*	0.87, (0.60, 0.96)

	Pre (mean ± SD)	Post (mean ± SD)	*p*	Rank Biserial Correlation (95% CI)
Number of bimanual activities (out of 27)	19.0 ± 6.5	20.8 ± 6.0	0.009*	0.56, (0.03, 0.86)

^*^ Significant *p* value (*p* ≤ 0.05)

#### Monitoring therapist field notes

During calls with the monitoring therapist, dyads spontaneously reported perceived improvements in one of their chosen ADLs on three occasions (once by each of three dyads), overall improvement in UL function (e.g., using the affected hand/arm more spontaneously) on four occasions (reported by three dyads), and feeling muscle tiredness (associated with doing meaningful UL therapy) on seven occasions (reported by six dyads).

## Discussion

This study evaluated the feasibility of implementation and the effectiveness of an 8-week home-based UL therapy gaming intervention, Bootle Blast, when used with 15 Costa Rican children with CP. Indicators of implementation were partially met, with 13 of 15 children completing the intervention. Of the 13, only three achieved their weekly playtime goal in at least six of eight intervention weeks. Technical barriers prevented three children from playing Bootle Blast for at least four days. Effectiveness was demonstrated with 11 children improving on at least one chosen ADL in the PQRS, while nine children achieved targeted gains (MCID) for the COPM.

### Implementation

In this study, we tested a novel approach where families were actively engaged in setting individualized aPTGs in line with their context, needs and capacity. Given that “lack of time” is one of the most cited reasons for non-adherence to home-based therapy programs [11, 40], this approach was selected to align with the principles of family-centred care [41] and to promote engagement by ensuring that the treatment plan was perceived as manageable [9, 42]. The design of Bootle Blast uniquely supports this approach in that the game rewards are linked to the child’s individualized aPTG. For example, a new mini-game programmatically unlocks each time a child reaches 17% of their total intervention aPTG to reward their progress and to renew novelty/excitement. This approach is counter to most previous studies in the field where the game rewards are not linked to individualized playtime goals, and where a uniform prescription is provided across participants [10, 15, 43].

Costa Rican families chose a mean aPTG (139 min per week) in phase 1 of the overarching project, prior to experiencing Bootle Blast [9], which was lowered to 60 min per week once they had been onboarded and had a better idea of the physical demands of playing the videogame. This aPTG was similar to a previous study of Bootle Blast in the Canadian context (56 min per week). Having a good understanding of what the game involved and how much play was recommended were important factors for dyads when determining aPTGs [9, 17]. Yet, it is not entirely clear if all families were aware of the difference between *active* and *total* playtime despite the monitoring therapist’s (DC) explanations (during screening, in a pre-intervention interview [9] and during the onboarding session) and the “mission time” clock displayed in the home screen. For most children, achieving their aPTG usually took twice as much time overall (e.g., achieving 20 min of active playtime took a child a total of 40 min). This could have affected the feasibility of achieving their weekly objective, as dyads might not have anticipated the additional time required to complete the weekly aPTG. This is an important consideration for future work when considering success indicators for the analysis of playtime.

When comparing playtimes among studies, it is also important to recognize that the intensity of movement may vary greatly between systems and between participants depending on their abilities and play styles. Costa Rican participants played on average 1.5 h/week total playtime. The playtimes reported in previous studies, largely conducted in high income countries, and considering only *total* playtime, varied from 0.3 to 7 h/week with a mean of 2.1 h/week [11]. Of note, the study in this scoping review [11] that reported the lowest weekly playtime (0.3 h/week) was an exception in that it did not provide participants with a weekly prescription or goal [44]. Across all studies in the review, playtime varied across participants and largely depended on time and family support [11], which was also the case in our study.

Interestingly, children with quadriplegia were among those who played the most. These children had determined and competitive personalities, regularly reported to be enjoying the game and experienced a high level of parental involvement and support during the intervention. Although most studies on rehabilitation videogaming interventions have focused on children with Gross Motor Function Classification System Levels I and II [10, 11], the success with which the children with quadriplegia in this study could engage with Bootle Blast when supported by parents, could suggests they should not be excluded. On the contrary, when families encountered life events that affected the amount of time and energy the child had to engage with Bootle Blast, playtime was usually low. For instance, during holiday breaks or weeks when children had additional schoolwork. Younger children who relied more on parental support also experienced decreased playtimes during weeks when there was a change in the routine involving the parent (e.g., parent was sick). This is also similar to our previous experience with Bootle Blast in the Canadian context, as when events disrupted the family schedule/routing, playtime decreased [17].

As in many other home-based videogame-based therapy studies [10, 11, 17], technical issues challenging the child’s sense of competence and autonomy (e.g., introducing feelings of frustration) were a barrier to engaging with Bootle Blast in Costa Rican children. While efforts were made to promptly address technical issues, insights gained from this study have informed system improvements for future interventions with Bootle Blast. For example, an automatic shut-down feature following 5–10 min of non-detected play has been added to the system to help prevent technical issues and improve the overall user experience. Additionally, improved in-game tutorials were added to mitigate any user frustration by enhancing the learnability of the videogame.

Conversely, other studies have shown that being able to choose when and how much to play and having scheduled follow ups with a monitoring therapist, motivated play with the videogame [10, 11, 17, 40]. Interestingly, Bootle Blast’s multiplayer mode was considered engaging by some, but not all participants. We speculate that there may be cultural aspects that contributed to differences from what has been reported on multi-player experiences in previous studies [11, 17]. This finding will be explored in depth in a future manuscript exploring the participants’ experiences with Bootle Blast.

Overall, not achieving weekly aPTGs does not necessarily indicate a lack of engagement with the intervention, as all children who experienced Bootle Blast completed the 8-week program and played most weeks (Appendix 2). The question of “dose” in rehabilitation interventions is complex and depends on many factors related to the individual, their goals, intensity and nature of practice [45]. As indicated in previous research [32] “more” is not always better, especially if results can be obtained with lower time commitments. Further research is needed to understand how Bootle Blast can best be “prescribed” and used in line with family routines and the characteristics of the child. However, key learnings from this study suggest:

- *Families should have a good understanding of the gaming program prior to deciding on a playtime goal. This could be facilitated by a short trial run with the game.*
- *Linking in-game rewards to playtime goals and intervention duration can be an effective strategy to sustain engagement over multiple weeks. For example, unlocking a new mini-game every 2 weeks can provide a more progressive experience and help with sustained motivation.*
- *Barriers to engagement in the Costa Rican context (e.g., time, life events, technical issues) are similar to those reported in the context of high-income countries, but with more ambiguity around the potential value of multiplayer modes that warrants further exploration.*
- *The use of therapy gaming technologies should be described relative to active and total playtime to better support comparison among studies and systems.*

### Effectiveness

Effectiveness was partially met with 11 of the 13 children who played Bootle Blast improving on the PQRS, and nine children achieving targeted gains (MCID) for the COPM. Some children who did not improve on the PQRS demonstrated better outcomes on the COPM, while others who did not reach the COPM’s MCID threshold showed important improvements on the PQRS. Both outcomes were selected to permit complementary measurement (objective and parent-reported) [24] of carryover of the Bootle Blast intervention into ADLs, contributing to a more comprehensive evaluation of overall change. Additionally, observations reported by the parents, such as increased spontaneous use of their child’s affected UL in other ADLs, along with higher scores on the BBT and CHEQ at post-intervention, further indicate the potential of skills transfer from Bootle Blast to everyday life function. These findings are consistent with those of a prior Bootle Blast study [17].

The Bootle Blast intervention appeared to lead to positive clinical outcomes with a modest commitment with respect to playtime. Factors influencing effectiveness of exergaming interventions may include the participants’ individual characteristics (e.g., MACS level), personal interactions with the system (e.g., *active* vs *passive* playtime) and level of intensity during gameplay [40, 43]. Understanding the nature and intensity of gameplay during exergaming interventions is necessary to enable comparisons among systems. In Bootle Blast, different mini-games target various aspects of UL function (Appendix 3), such as big joint movements (e.g., shoulder flexion) and fine motor skills (e.g., block manipulations). The seemingly high intensity of the Bootle Blast intervention (e.g., participants reporting feeling muscle fatigue after playing) along with the diversity in games and targeted movements (e.g., games with obstacle avoidance, mixed-reality.), may have contributed to achieving UL motor improvements within playtimes that are lower than the average suggested by the literature (i.e., 14–25 h. of practice for a treatment block) [40, 43].

It is also possible that the potential for improvement was high for this group of Costa Rican participants who had received little to no UL rehabilitation previously [9]. This aligns with prior work in the field which has shown some indication that greater functional gains are realized by children with lower baseline functional scores [43, 46]. While positive changes were associated with the overall intervention, it should be noted that Bootle Blast was just one component. Other factors within this study design, such as the video calls from the monitoring therapists and the PQRS weekly testing could have influenced the clinical outcomes. Increases in the COPM scores indicate parents were conscious of their child’s improved abilities, and this awareness may have been heightened due to weekly filming of these activities. Despite parents being blinded to their pre-intervention COPM scores, the focus on hand and arm function, an area previously overlooked, may have acted as an intervention itself. While the inability to attribute causality should be acknowledged, it should not undermine the importance of the findings, and is in part mitigated by the establishment of baseline performance via the weekly PQRS testing in phase A. Additionally, while not related to their UL goals, it is of note that participants 10 and 12 were receiving physical therapy treatment in preparation for lower limb surgery.

Moving forward, the addition of in-depth qualitative data from the participants’ experiences with the intervention (phase 3 of the overarching project, which will be published as a separate manuscript) will facilitate a more holistic understanding of the level of engagement, enjoyment, and the perceived value of this intervention for improving functionality in UL bimanual activities [11, 40]. Furthermore, while the small sample size of this study precluded conclusions regarding the extent to which sociodemographic factors (rural vs urban, socioeconomic status) impacted outcomes, this is an important area for future research.

Key learnings pertaining to the Bootle Blast intervention’s effectiveness that emerged from this study include:

- *Overall positive clinical outcomes were achieved with modest time commitments, demonstrating carryover to ADLs.*
- *Characteristics of the intervention (e.g., presence of a monitoring therapist), the child (e.g. baseline functional levels) and the family environment (e.g. level of support) should be considered when interpreting the effectiveness of therapy gaming interventions.*
- *The nature and intensity of the gameplay, in addition to playtime alone, warrants additional attention when assessing effectiveness of exergaming interventions.*

## Limitations

The remote nature of this work introduced certain challenges, including some technical difficulties (e.g., losing Wi-Fi during a call), potential disruptions in children’s attention while doing clinical assessments over Zoom at home, and limited the selection of UL clinical assessments to those that could be virtually administered. For example, assessments like the Assisting Hand Assessment [47] which measures bimanual hand use (clinician-scored) were deemed too complex to be assessed virtually. Additionally, some goals established during phase 1 of the overarching project [9] were found to be less compatible with video observation (e.g., improving grasp when using a walker, which was the case for participant 7). While connecting virtually facilitated access to data and inclusion of “hard-to-reach” participants [48], supported the family-centered approach, and allowed real-world implementation of the intervention, future research should ensure that ADLs selected for virtual assessment are conducive to measurement in that format.

Although we aimed to conceal the randomization process to enhance the study's validity, modifications were necessary for four families to facilitate participation. For some children, waiting to play the video game was challenging, and the lengthy baseline was causing family stress. For one dyad, the baseline was shortened by a week to ensure the child could complete the intervention phase before a week-long commitment that arose. While these modifications could potentially impact the study's validity, it is important to note that they were family- rather than researcher-instigated, thereby reducing the potential for selection bias.

The small sample size (n = 13) affected the interpretation of the effectiveness outcome for the COPM (i.e., 6% of participants under the targeted outcome level represents 0.78 of a child) and limited in-depth, multivariate exploration of the potential factors influencing clinical outcomes. The single case experimental design was however considered a strength that maximized rigour and internal validity with the small and heterogeneous sample. Sample size may have also affected the reliability statistics for the PQRS. For one participant, a 9-point difference in scores between raters in the “task completion” domain influenced the reliability score for ADL2 at T1. This was not the case for the “quality of movement domain”, which was scored consistently across raters. While the reason for this discrepancy could not be ascertained (potentially a typo or possibly what indicated successful completion for this goal was not clearly communicated), it is important to note this was an isolated event across the sample.

## Conclusions

Bootle Blast has the potential to support improvements in overall motor performance of the UL during ADLs, when implemented in a real-world environment in Costa Rican children with CP. Using strategies (e.g., trial run with the Bootle Blast system, additional education to participants to facilitate setting playtime goals) to ensure a good fit between the intervention, the family and the child are key for successful implementation. Positive changes in measurement scores with modest playtime suggest that UL motor improvement does not necessarily depend on the amount of time spent playing, but may be influenced by other variables such as the nature and intensity of the practice, baseline function, and the therapy goals selected. Exergaming home interventions, like Bootle Blast, can provide opportunities for home therapy for children with CP, especially for those living in low-income families, rural areas, and remote communities, and as a complement to traditional clinician-led interventions.

## Supplementary Information

Supplementary Material 1

Supplementary Material 2

Supplementary Material 3

Supplementary Material 4

Supplementary Material 5

## Data Availability

Numeric non-identifiable data regarding the PQRS and game logs are provided within this manuscript and its supplementary information files. Datasets concerning the COPM, CHEQ, BBT and field notes from weekly phone calls are not publicly available as this was not stated in the consent form provided to participants, but are available from the corresponding author on reasonable request.

## Abbreviations

CP: Cerebral Palsy
UL: Upper limb
ADLs: Activities of daily living
aPTG: Active playtime goal
PQRS: Performance Quality Rating Scale
COPM: Canadian Occupational Performance Measure
BBT: The Box and Blocks Test
CHEQ: Children’s Hand-use Experience Questionnaire
MACS: Manual Ability Classification System
MCID: Minimally clinical importance difference
SMD: Standard mean difference
PND: Percentage of non-overlapping data
