# Supplementary figures and images for: Feasibility of a home-based home videogaming intervention with a family-centered approach for children with cerebral palsy: a randomized multiple baseline single-case experimental design
Source: J Neuroeng Rehabil. 2024 Sep 4;21:151. doi: 10.1186/s12984-024-01446-2 (PMC11373410; doi:10.1186/s12984-024-01446-2)

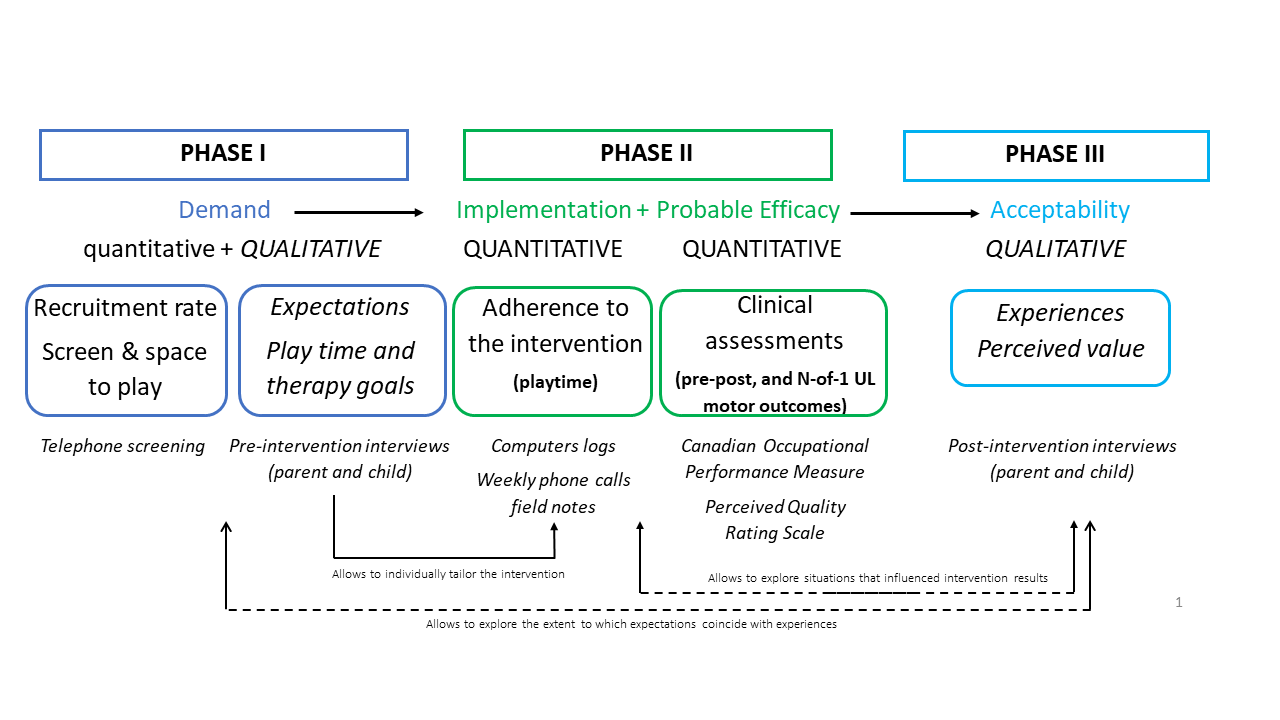

Supplement: Supplementary file 1 — Supplementary Material 1 [file 12984_2024_1446_MOESM1_ESM.png]
