# Supplementary material for: Feasibility of a home-based home videogaming intervention with a family-centered approach for children with cerebral palsy: a randomized multiple baseline single-case experimental design
Source: J Neuroeng Rehabil. 2024 Sep 4;21:151. doi: 10.1186/s12984-024-01446-2 (PMC11373410; doi:10.1186/s12984-024-01446-2)
Supplement: Supplementary file 2 — Supplementary Material 2 [file 12984_2024_1446_MOESM2_ESM.pdf]

Appendix 2. Individual active playtime by intervention week

Note: Two different y axes (minutes) are presented among participants to better show inter session variability

Dotted line refers to trendline. Y axis 0-100.

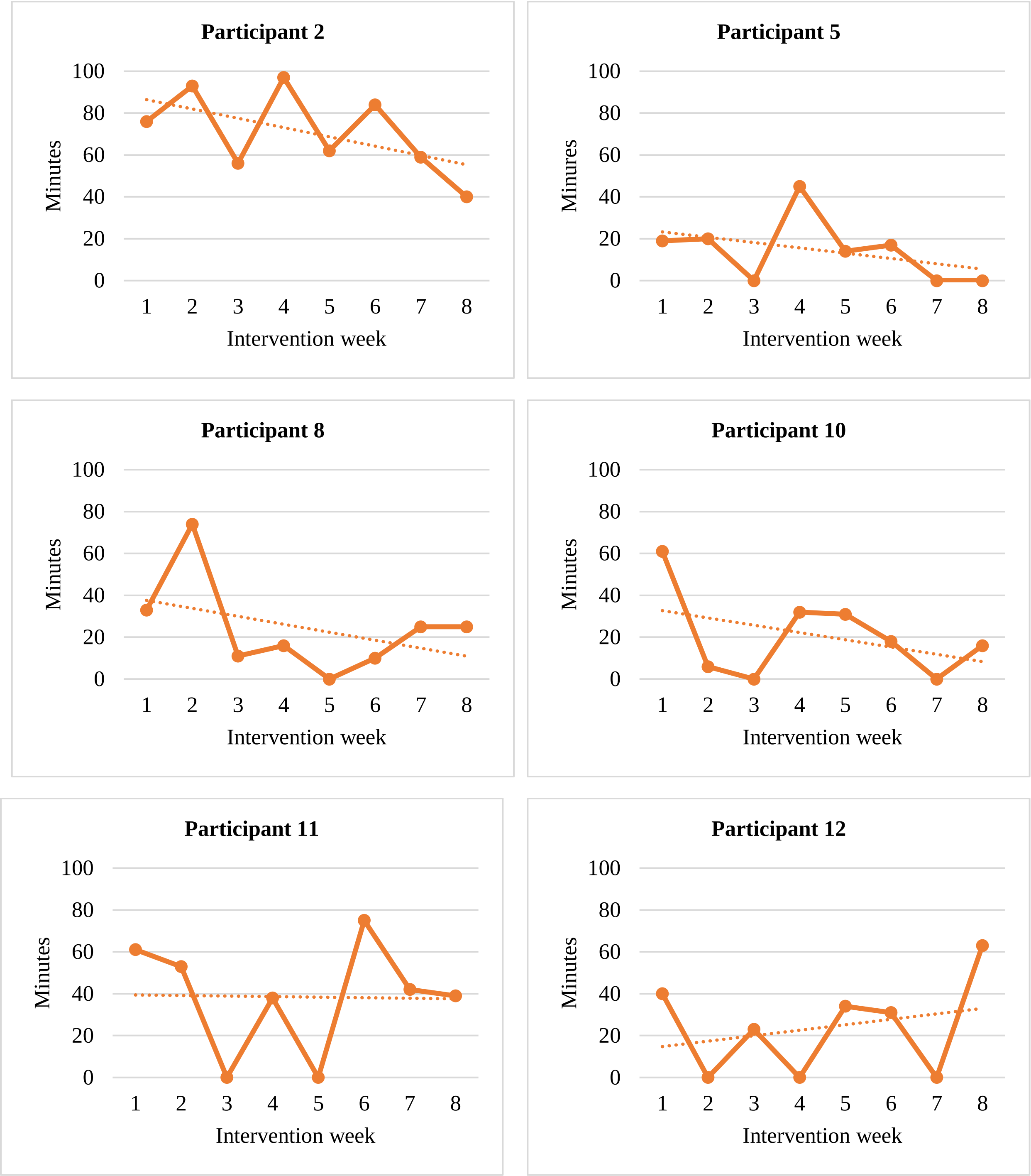

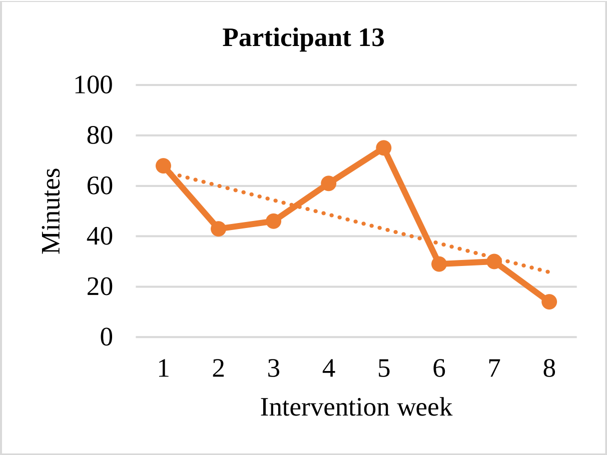

Dotted line refers to trendline. Y axis 0-280.

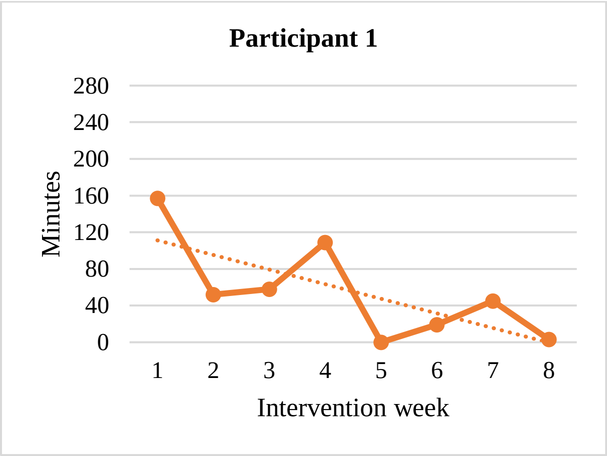

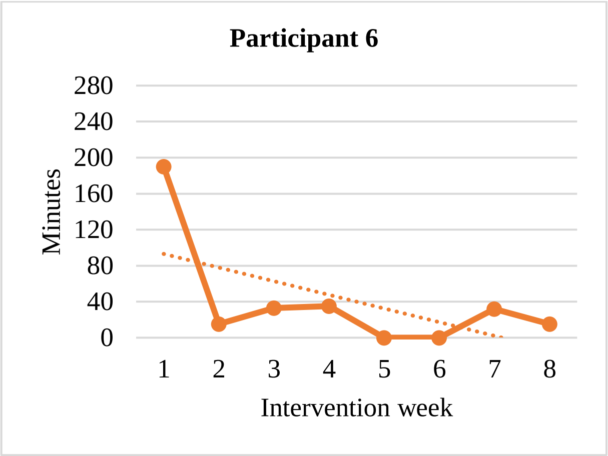

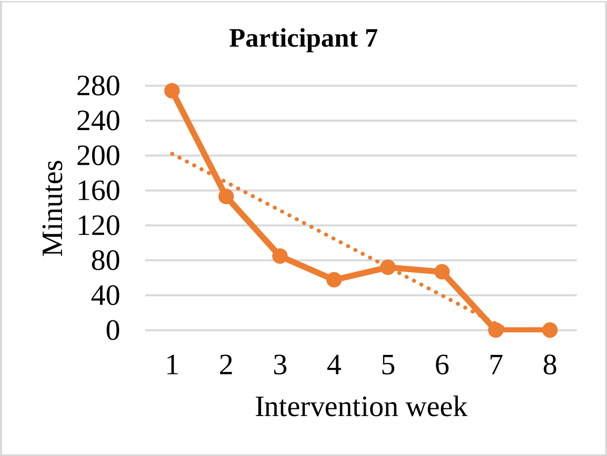

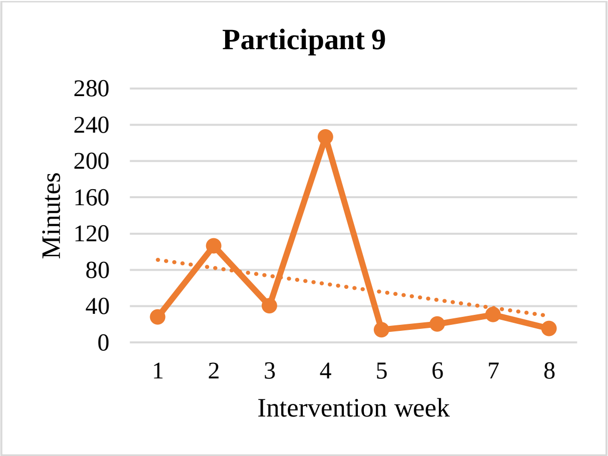

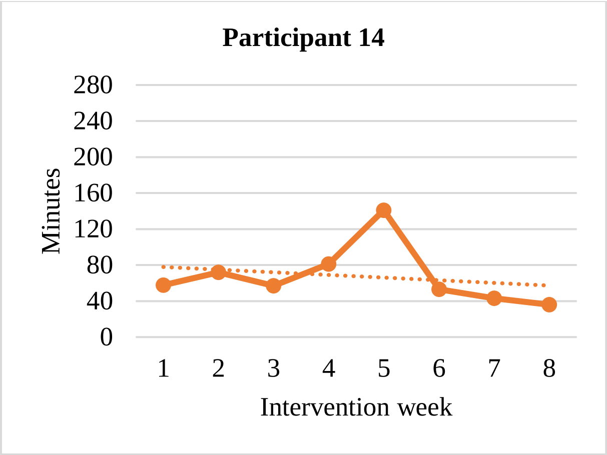
Participant 14
280
240
200
160
120
80
40
0
Minutes
1 2 3 4 5 6 7 8
Intervention week

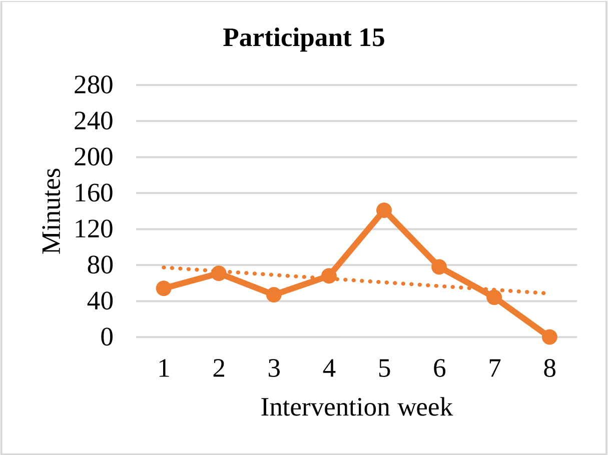
Participant 15
280
240
200
160
120
80
40
0
Minutes
1 2 3 4 5 6 7 8
Intervention week
