# Supplementary material for: Feasibility of a home-based home videogaming intervention with a family-centered approach for children with cerebral palsy: a randomized multiple baseline single-case experimental design
Source: J Neuroeng Rehabil. 2024 Sep 4;21:151. doi: 10.1186/s12984-024-01446-2 (PMC11373410; doi:10.1186/s12984-024-01446-2)
Supplement: Supplementary file 3 — Supplementary Material 3 [file 12984_2024_1446_MOESM3_ESM.pdf]

Supplementary Appendix 1. Table 12. Mini-games Summary

Mini-game	Game play	Targeted movements	Toys needed	Player options
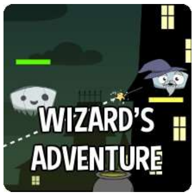	Player defends a castle against a wave of ghosts. When you see a Ghost Bootle approaching, move your arm up to the side (i.e. shoulder abduction) to aim the Wizard's wand. Hold your arm in position until the Ghost Bootle is zapped away. Extend your elbow for extra range. Rest your arm by your side to recharge your wand in the magic pot.	Shoulder abduction<br>Elbow extension<br>Plays with most affected upper limb	None	Single player<br>Multiplayer co-operative
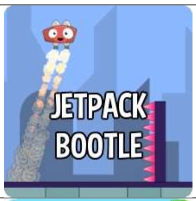	Player moves targeted arm up and down in front of them (e.g. shoulder flexion) to avoid obstacles in this endless flyer game.	Shoulder flexion<br>Plays with most affected upper limb	None	Single player
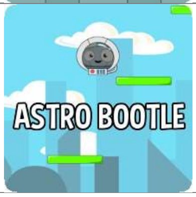	Player leans left and right to jump from platform to platform, avoiding aliens and collecting power-ups in this endless jumper game.	Trunk lean	None	Single player
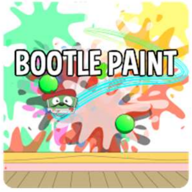	Player reaches for moving coloured targets in order to splatter them on the painter's canvas, and avoids "bombs". Player must switch hands when prompted.	Shoulder abduction/flexion<br>Cross body reach<br>Elbow extension<br>Plays with both upper limbs	None	Single player
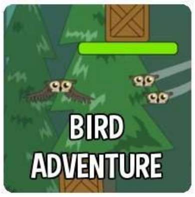	Player explores an open-ended world using a "flapping" arm movement to travel as a Bootle Bird. There are many interactive elements to the map, and 9 special tasks to complete in order to collect all of the stars. Flapping with the left arm moves the bird right and vice versa.	Shoulder abduction<br>Bilateral coordination	None	Single player

Appendix 1 (continue)

Mini-game	Game play	Targeted movements	Toys needed	Player options
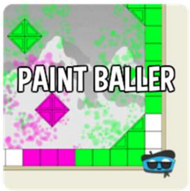	Player performs a “clap” motion to fire paint balls and colour fill the most territory in this competitive game. To fire the paint ball, make sure you open your arms wide enough, and then clap! The on-screen hand icons will turn white when you are ready to fire.	Bilateral coordination; bringing hands to mid line.	None	Single player<br>Multiplayer competitive
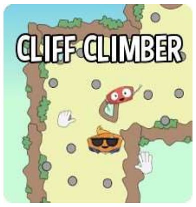	Player reaches to grab handholds and climb a mountain, collecting Bootles as they go. You can move just one hand at a time while your other hand remains anchored to the last handhold position. Hover over a handhold to grasp it. Then reach for the next handhold with your other hand.	Bilateral coordination<br>Cross body reach<br>Elbow extension<br>Shoulder abduction/flexion	None	Single player<br>Multiplayer competitive
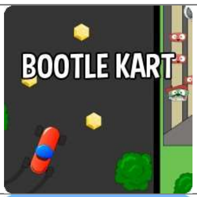	Player steers an imaginary (or real) steering wheel and navigates their kart to avoid obstacles and collect gems.	Bilateral coordination	None	Single player<br>Multiplayer competitive
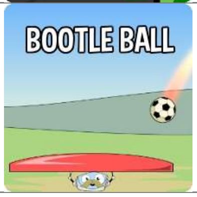	Player rotates a baton (wrist supination) to control the position of a platform and keep a bouncing ball in the air.	Wrist supination and pronation<br>Plays with most affected upper limb	Red-green baton	Single player<br>Multiplayer competitive
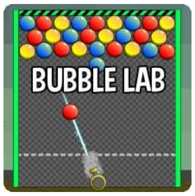	Player picks up and shows the camera a Mega Blok of the colour they want to use. Hold the Mega Blok up in front of you above your shoulder. Make sure that the camera can see the block clearly. Try to connect 3 bubbles of similar colour to pop them.	Grasp and release	Red, blue, yellow Mega Bloks	Single player
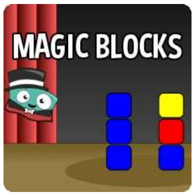	Player replicates on-screen coloured towers using real- life Mega Bloks. Once you have built the tower, hold it up in front of you above your shoulder in order for the game to score your tower. Make sure that the camera can see the block clearly.	Grasp, manipulation and release	Red, blue, yellow, green Mega Bloks	Single player<br>Multiplayer competitive

Appendix 1 (continue)

Mini-game	Game play	Targeted movements	Toys needed	Player options
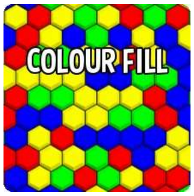	Player picks up and shows the camera a Mega Blok of the colour they want to play. The object of the game is to colour fill the grid in as few moves as possible. Hold the block up high in front of you and make sure that the camera can see the block clearly.	Grasp and release	Red, blue, yellow, green Mega Bloks	Single player Multiplayer competitive
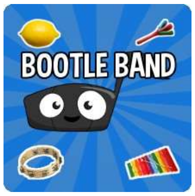	Player manipulates real-life instruments to create a Bootle Band. Game tasks involve "busking" to collect coins (level 2); recording songs (level 3); and playing in a concert (level 4).	Grasp, manipulation, and release	Tambourine Maraca Castanet Xylophone for levels 2+	Single player<br>Multiplayer collaborative
