# Supplementary material for: Feasibility of a home-based home videogaming intervention with a family-centered approach for children with cerebral palsy: a randomized multiple baseline single-case experimental design
Source: J Neuroeng Rehabil. 2024 Sep 4;21:151. doi: 10.1186/s12984-024-01446-2 (PMC11373410; doi:10.1186/s12984-024-01446-2)
Supplement: Supplementary file 4 — Supplementary Material 4 [file 12984_2024_1446_MOESM4_ESM.pdf]

Appendix 4. Performance Quality Rating Scale (PQRS) Single subject design visual analysis per participant

For all graphs:

Figure represents the *PQRS scores across baseline and intervention phases* for each chosen activity of daily living (ADL).

Slope trend line is represented by the red line at baseline, and dashed line at intervention.

Blue line marks the amount of points at B which surpased the highets score at A *(Percentage of non overalpping data [PND]).*

Yellow lines indicates the mean score at each phase respresenting *slope level change*.

Participant 1

ADL 1

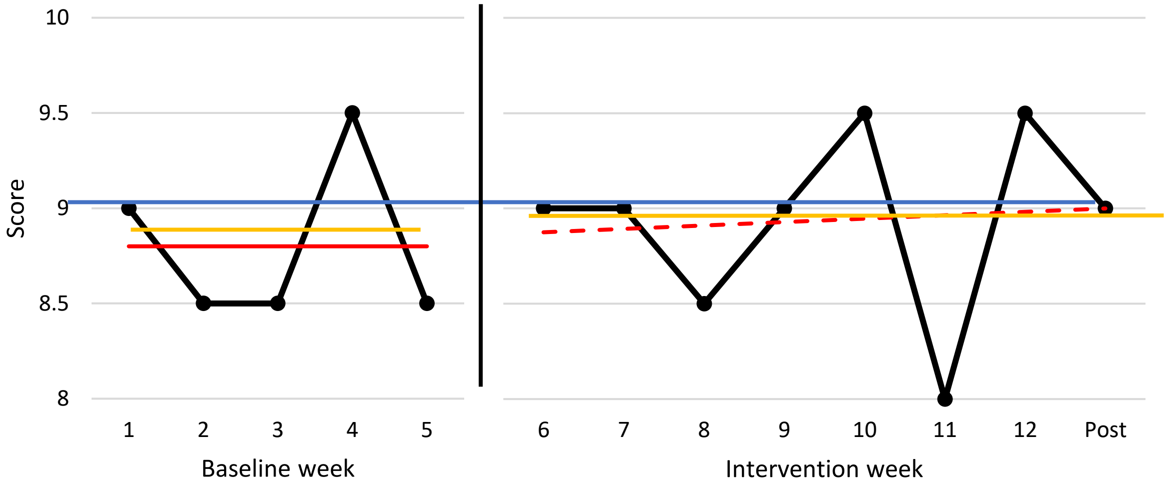

ADL	Improving ability to eat finger foods
PQRS improvement	0 pts
Standard mean difference	0.31 pts
PND	0% (*p=1*)

ADL 2

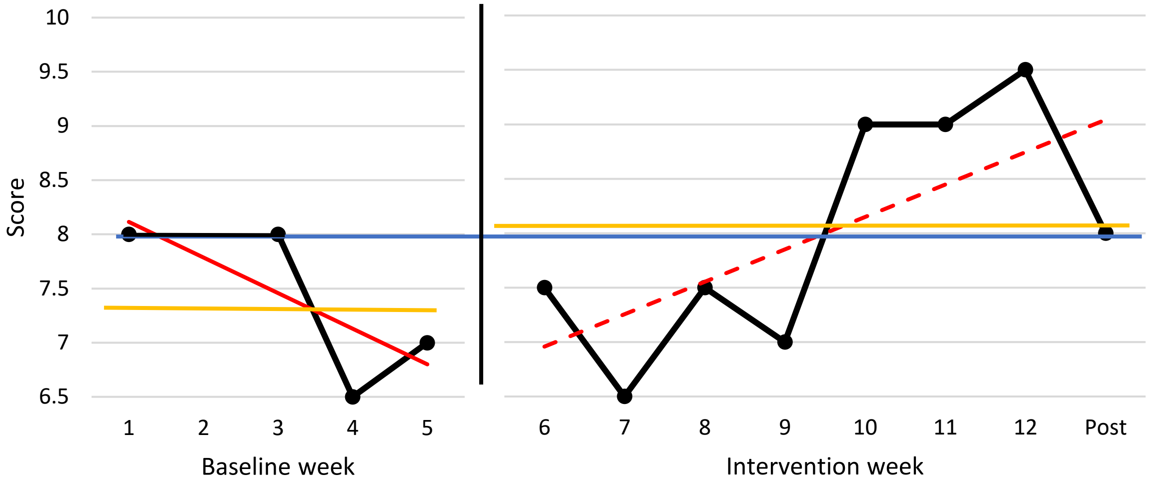

Missing data at baseline week 2.

ADL	Buttoning up a large button on a verst
PQRS improvement	1.50 pts
Standard mean difference	0.83 pts
PND	37.50 % (*p=0.20*)

Participant 2

ADL 1

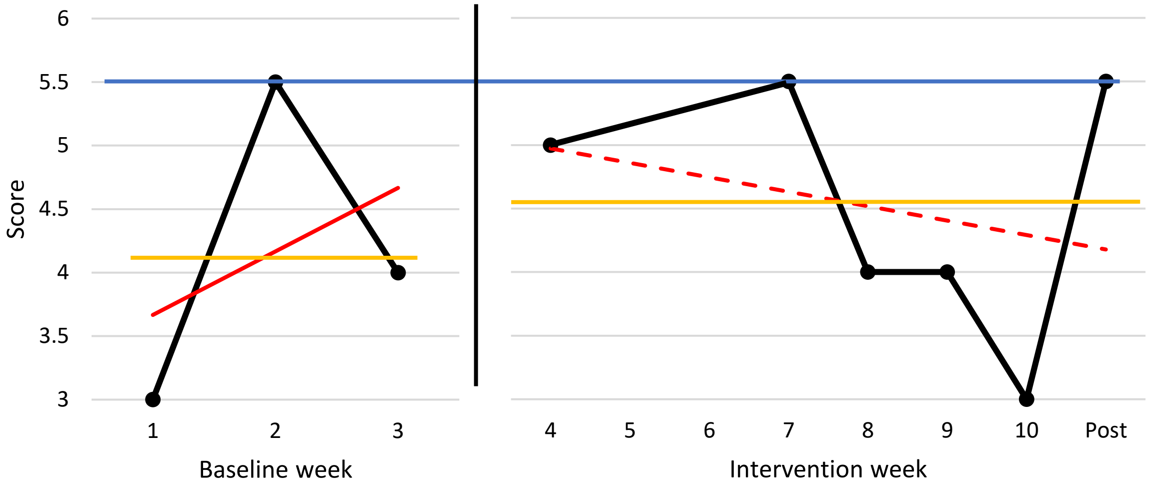

Missing data at interventions weeks 5 and 6.

**ADL**	**Buttoning up the school shirt**
PQRS improvement	0 pts
Standard mean difference	0.26 pts
PND	0% (*p=1*)

ADL 2

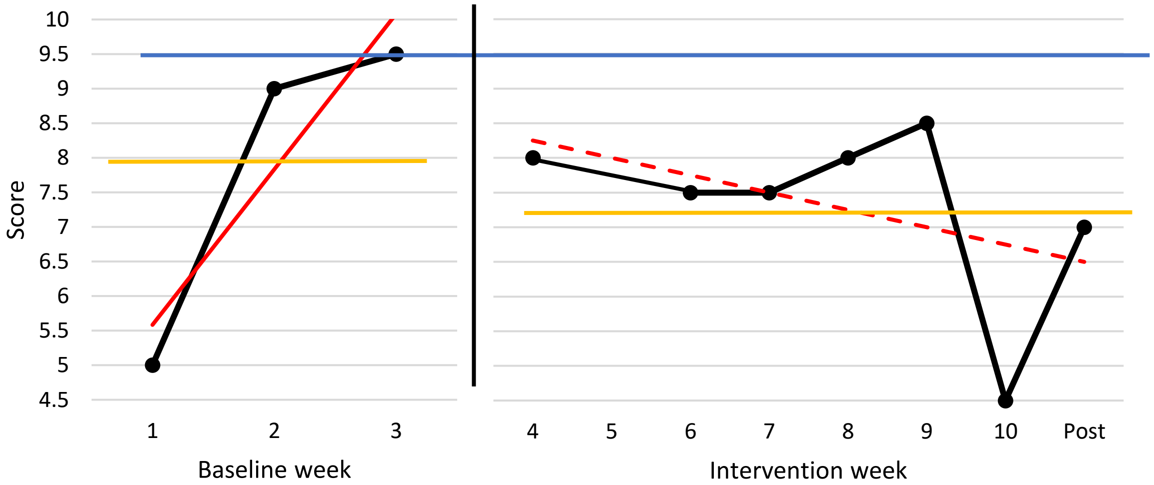

Missing data at intervention week 5.

**ADL**	**Improving ability to type in the computer**
PQRS improvement	0 pts
Standard mean difference	-0.22 pts
PND	0% (*p=1*)

Participant 5

ADL 1

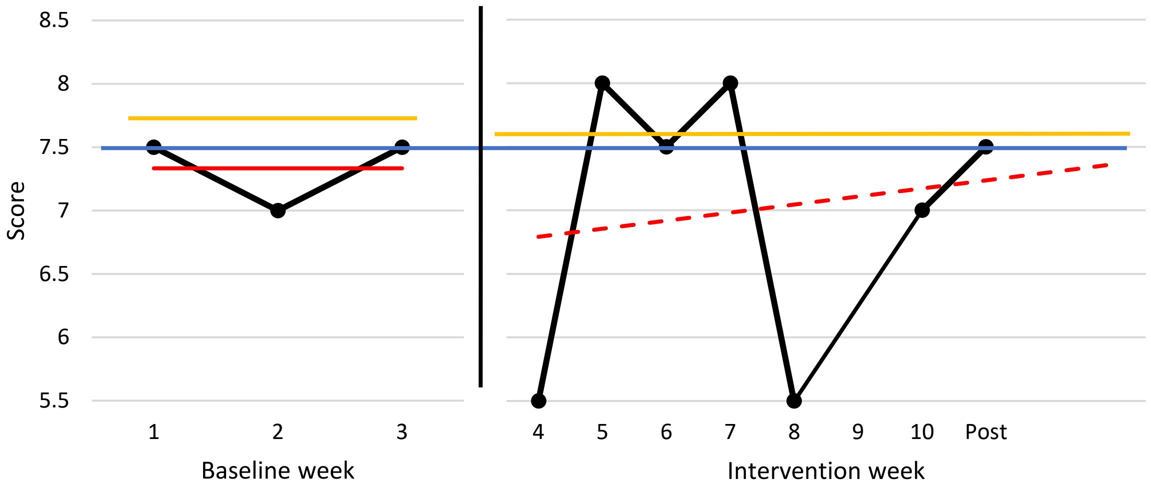

Missing data at intervention week 9.

**ADL**	**Putting socks independently**
PQRS improvement	1 pts
Standard mean difference	-1.15 pts
PND	23.57% (*p=0.38*)

ADL 2

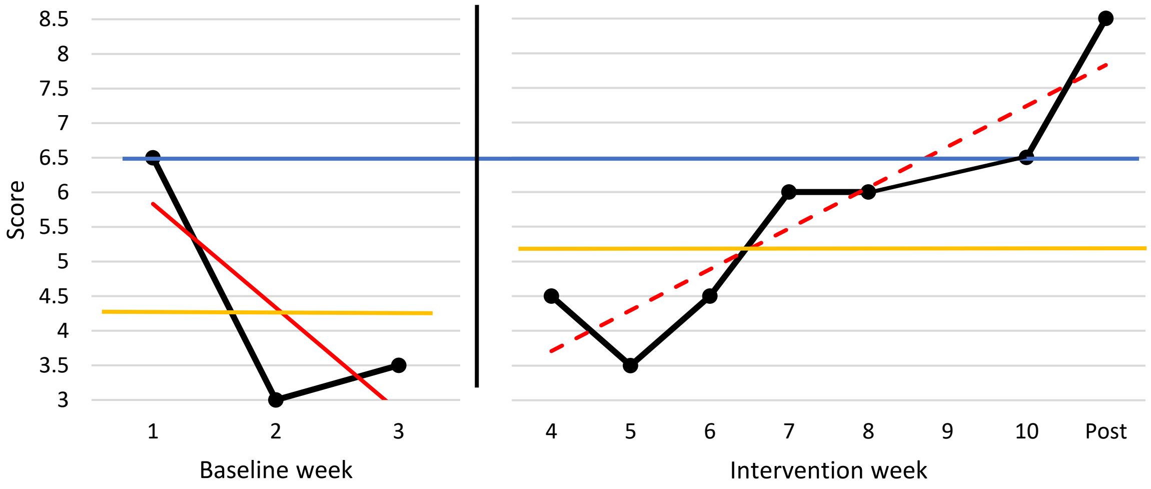

Missing data at intervention week 9.

**ADL**	**Improving ability to type in the computer**
PQRS improvement	2 pts
Standard mean difference	0.69 pts
PND	14.3 % (*p=0.57*)

Participant 6

ADL 1

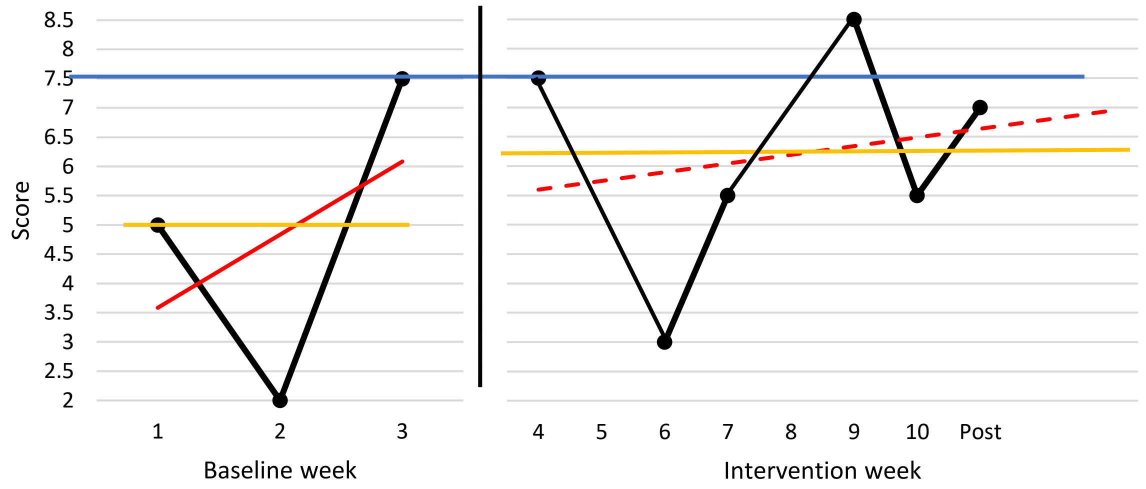

Missing data at intervention weeks 5 and 8.

**ADL**	**Buttoning up the school shirt**
PQRS improvement	1 pts
Standard mean difference	0.48 pts
PND	16.67% (*p=0.54*)

ADL 2

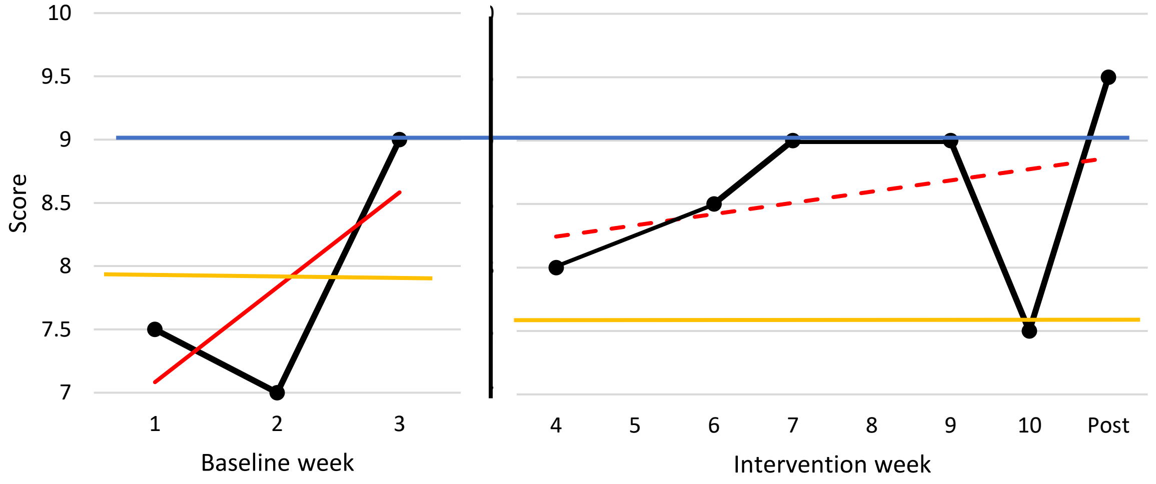

Missing data at intervention weeks 5 and 8.

**ADL**	**Tying shoelaces**
PQRS improvement	0.5 pts
Standard mean difference	-0.35pts
PND	16.67% (*p=0.54*)

Participant 7

ADL 1

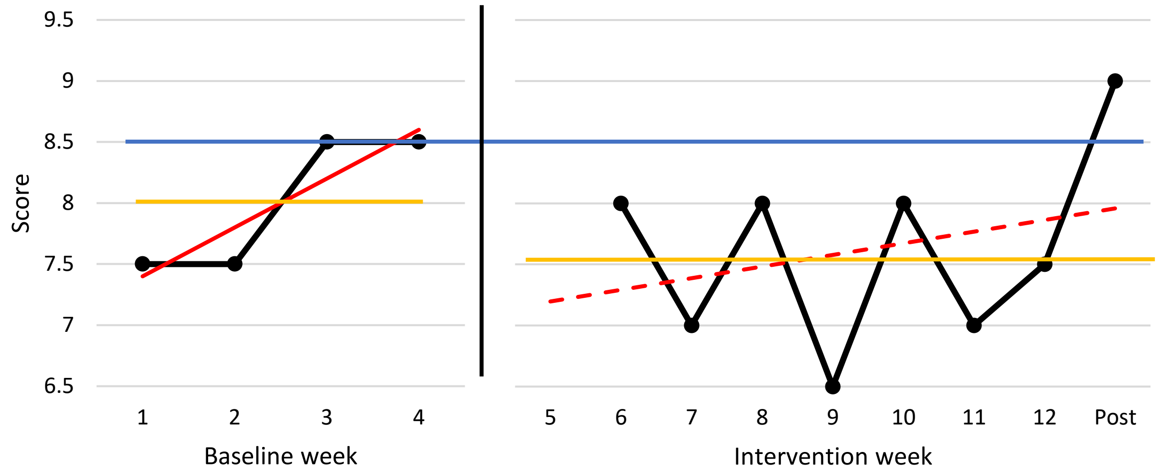

**ADL**	**Improving hand grasp (affected) when using the walker**
PQRS improvement	0 pts
Standard mean difference	-0.99 pts
PND	0% (*p=1*)

ADL 2

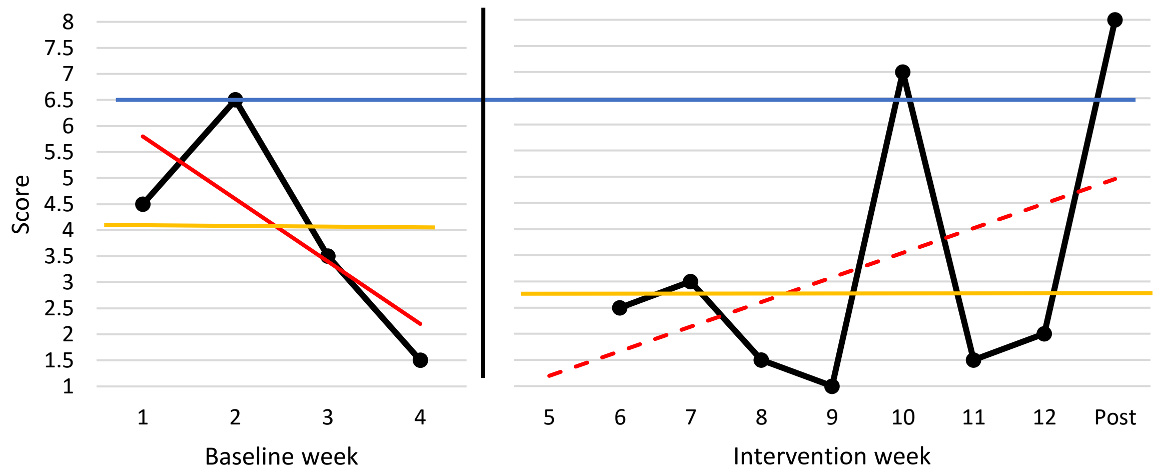

**ADL**	**Holding on to a loose paper, to be able to write on it**
PQRS improvement	0.5 pts
Standard mean difference	-0.65 pts
PND	14.29% (*p=0.5*)

Participant 8

ADL 1

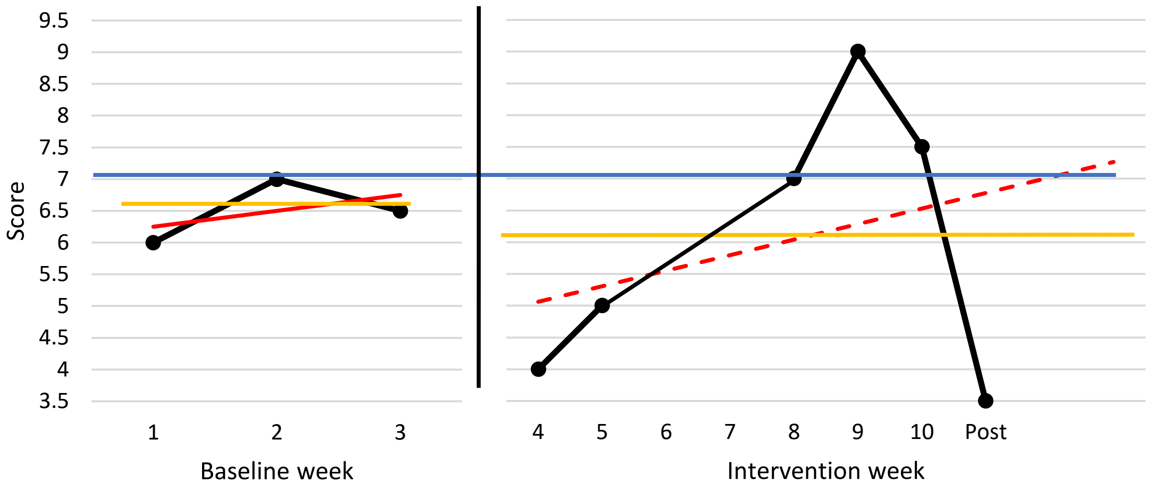

Missing data at intervention weeks 6 and 7.

**ADL**	**Buttoning up school shirt**
PQRS improvement	0.5 pts
Standard mean difference	-1 pts
PND	33% (*p=0.33*)

ADL 2

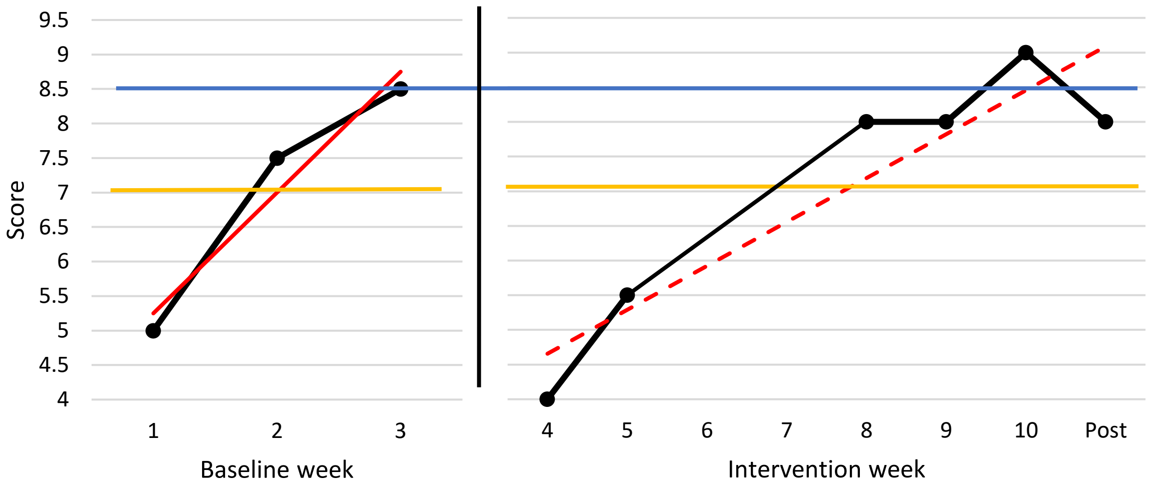

Missing data at intervention weeks 6 and 7.

**ADL**	**Cutting with knife and fork**
PQRS improvement	0.5 pts
Standard mean difference	0.05 pts
PND	16.67% (*p=0.54*)

Participant 9

ADL 1

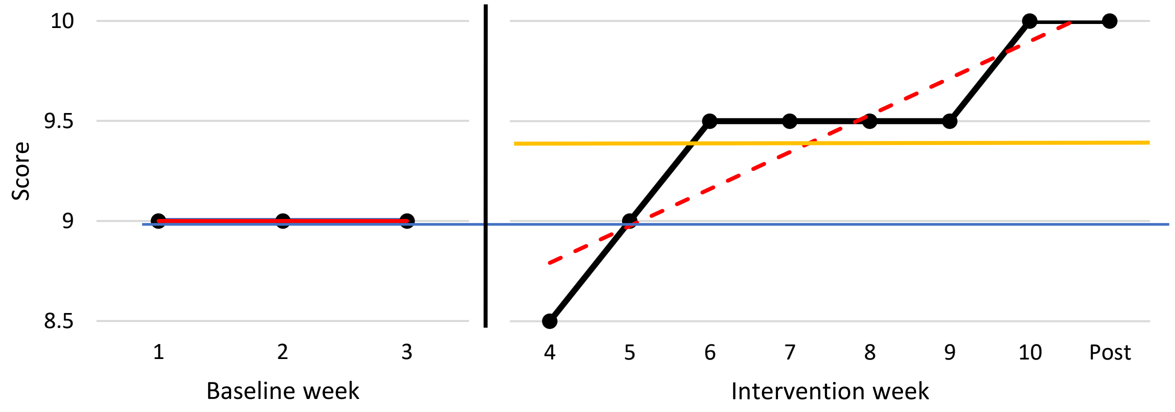

**ADL**	**Improving ability to put on the school shirt (i.e., buttoning up, fixing the collar, putting shirt inside pants).**
PQRS improvement	1 pts
Standard mean difference	unable to calculate as the standard deviation of the mean in phase A is 0 (i.e., data suggests a high SMD)
PND	75% (*p=0.05*)

ADL 2

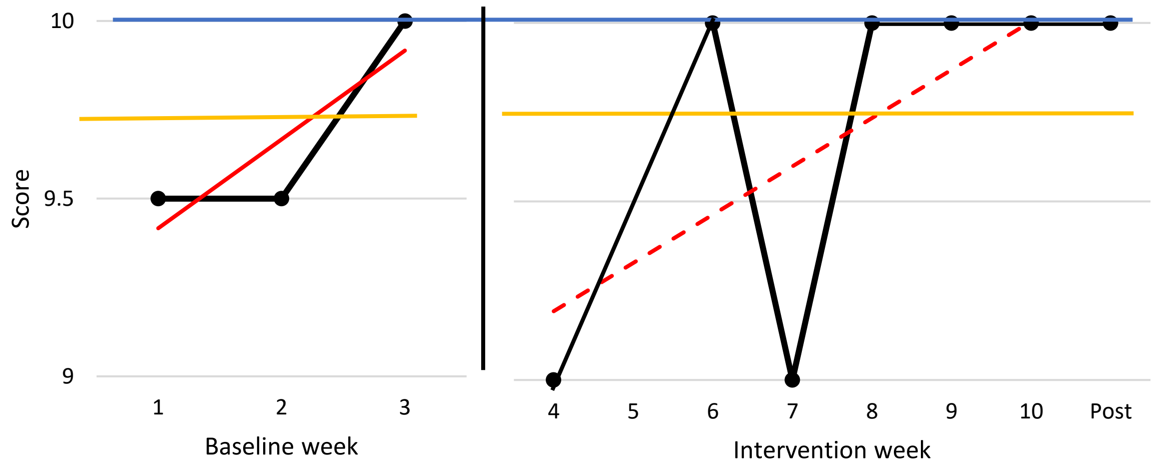

Missing data at intervention week 5.

**ADL**	**Tying shoelaces**
PQRS improvement	0 pts
Standard mean difference	0.16 pts
PND	0% (*p=1*)

Participant 10

ADL 1

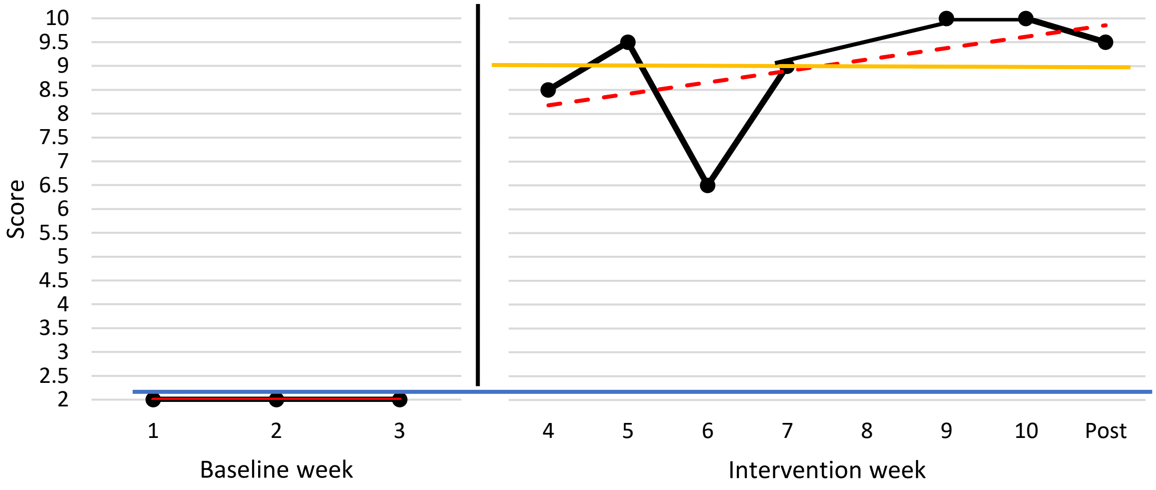

Missing data at intervention week 8.

**ADL**	**Button jeans**
PQRS improvement	8 pts
Standard mean difference	unable to calculate as the standard deviation of the mean in phase A is 0 (i.e., data suggests a high SMD)
PND	100% (*p=0.005)*

ADL 2

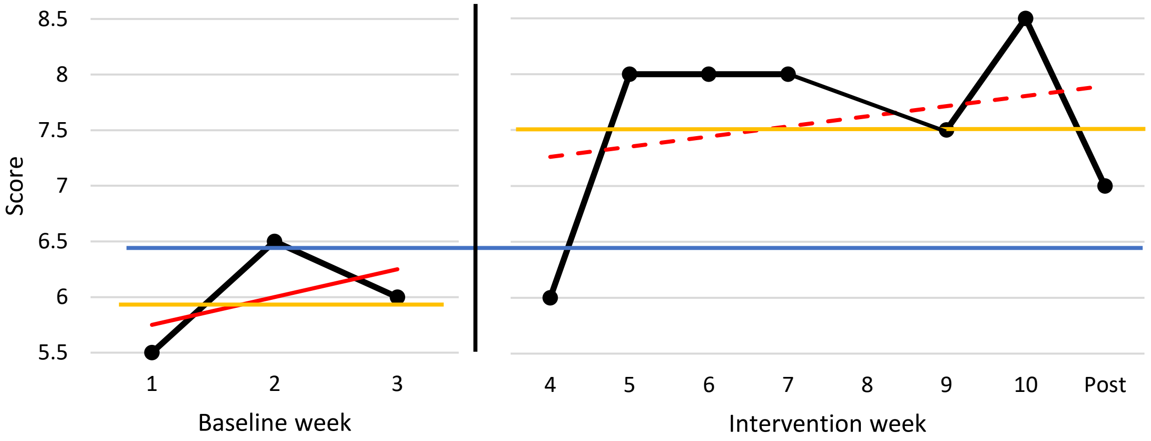

Missing data at intervention week 8.

**ADL**	**Improving ability to type in the computer**
PQRS improvement	2 pts
Standard mean difference	3.14 pts
PND	85.71% (*p=0.023*)

Participant 11

ADL 1

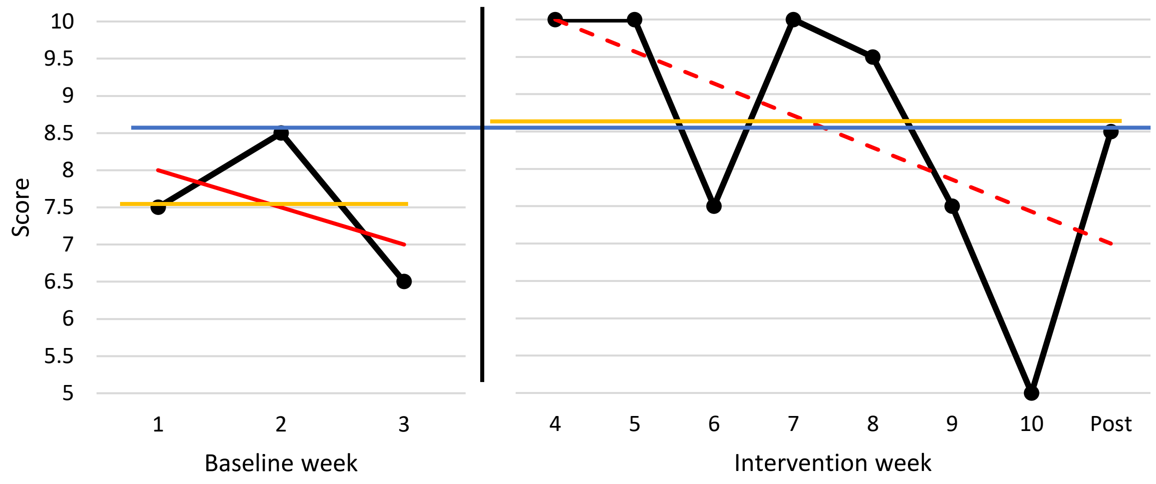

**ADL**	**Cutting with knife and fork**
PQRS improvement	1.5 pts
Standard mean difference	1 pts
PND	50% (*p=0.17*)

ADL 2

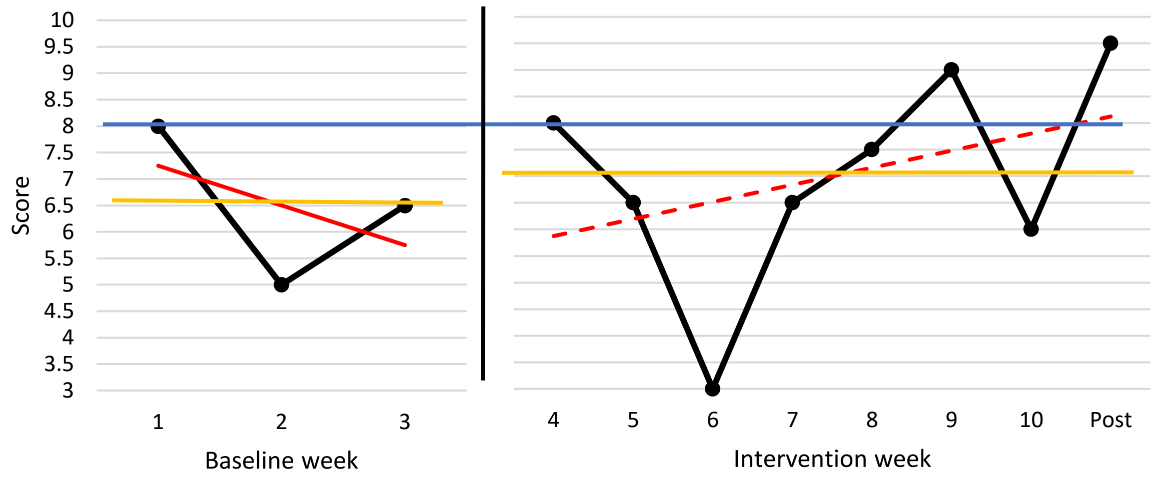

**ADL**	**Tying shoelaces**
PQRS improvement	1.5 pts
Standard mean difference	0.33 pts
PND	25% (*p=0.43*)

Participant 12

ADL 1

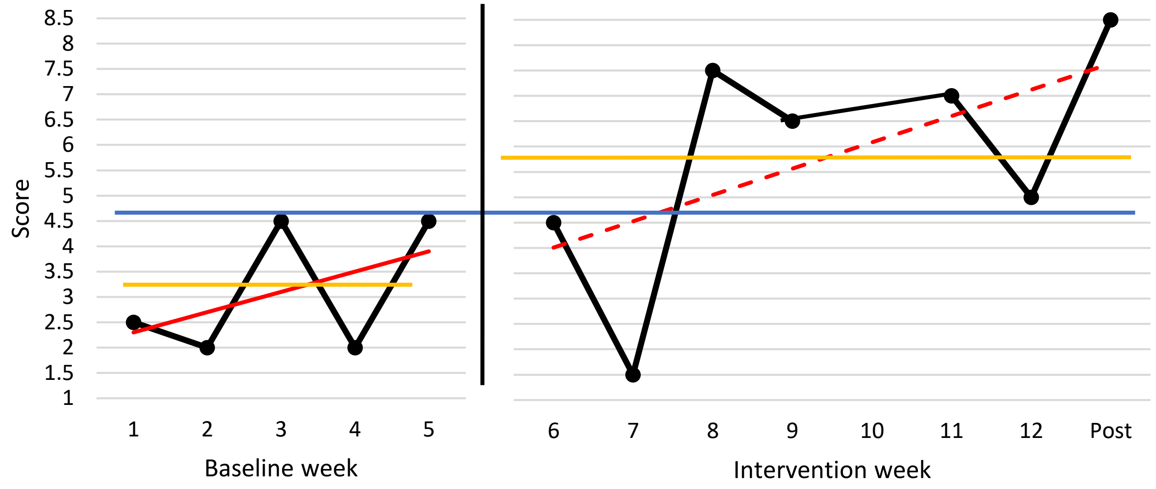

Missing data at intervention week 10.

**ADL**	**Buttoning up the school shirt**
PQRS improvement	4 pts
Standard mean difference	2.08 pts
PND	71.43% (*p=0.018*)

ADL 2

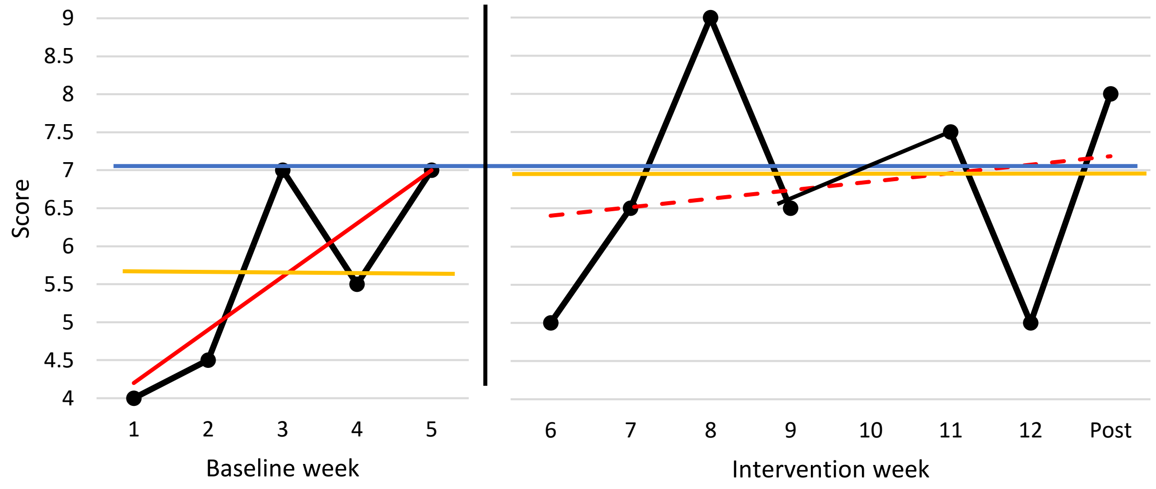

Missing data at intervention week 10.

**ADL**	**Putting socks on**
PQRS improvement	3.5 pts
Standard mean difference	2.25 pts
PND	42.86% (*p=0.12*)

Participant 13

ADL 1

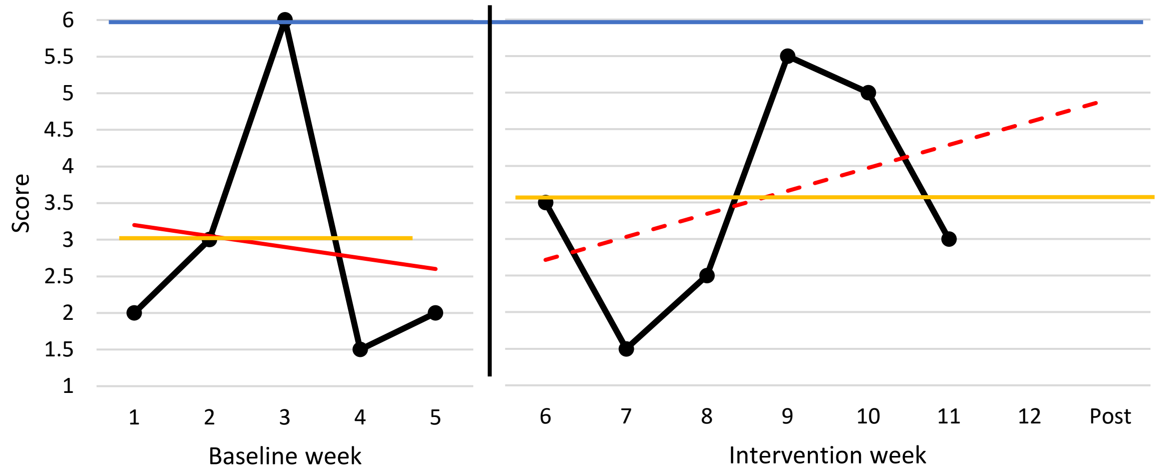

Missing data at intervention week 12 and post.

**ADL**	**Cutting with knife and fork**
PQRS improvement	0 pts
Standard mean difference	0.33 pts
PND	0% (*p=0.21*)

ADL 2

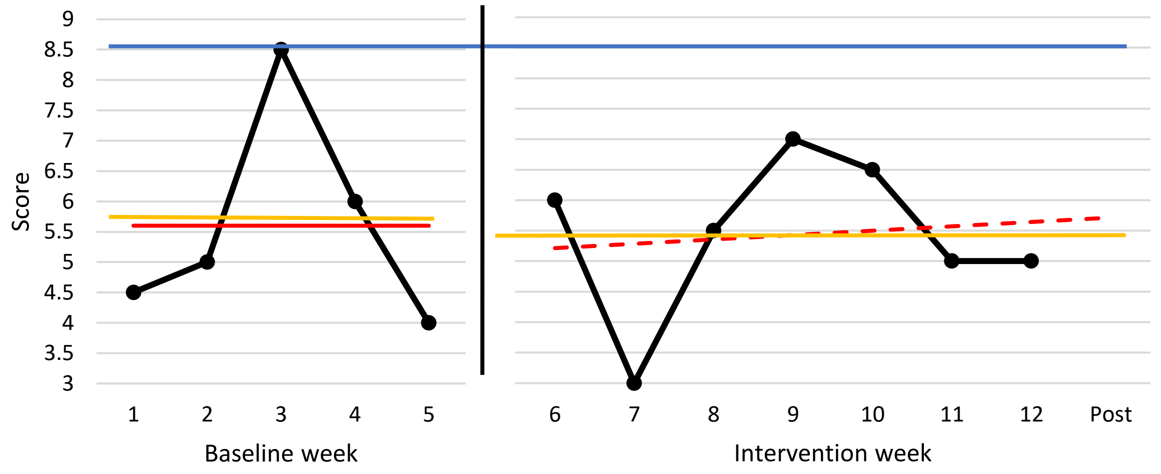

Missing data at post.

**ADL**	**Taking out small items (with dominant hand) from a bag (while holding it with affected hand)**
PQRS improvement	0 pts
Standard mean difference	-0.09 pts
PND	0% (*p=1*)

Participant 14

ADL 1

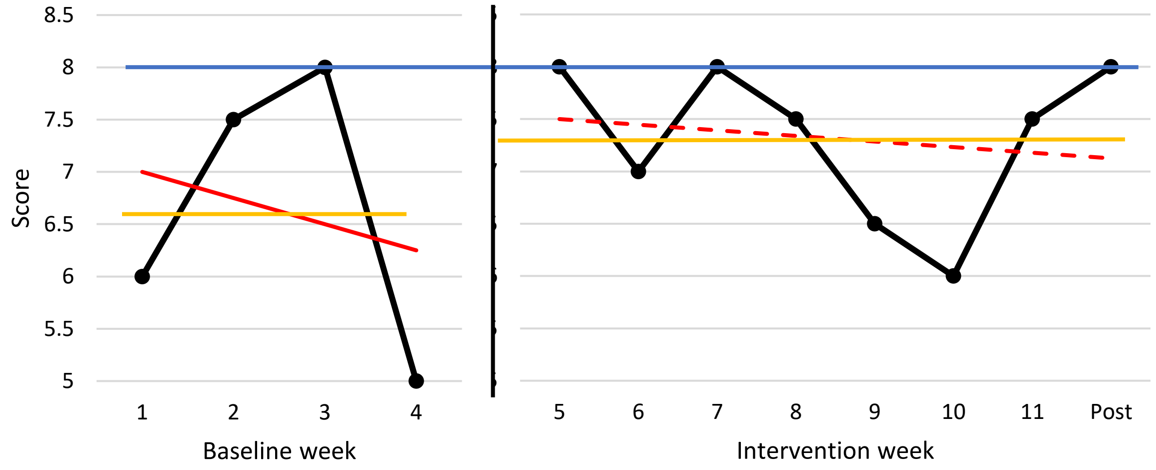

**ADL**	**Cutting with knife and fork**
PQRS improvement	0 pts
Standard mean difference	0.50 pts
PND	0% (*p=0.35*)

ADL 2

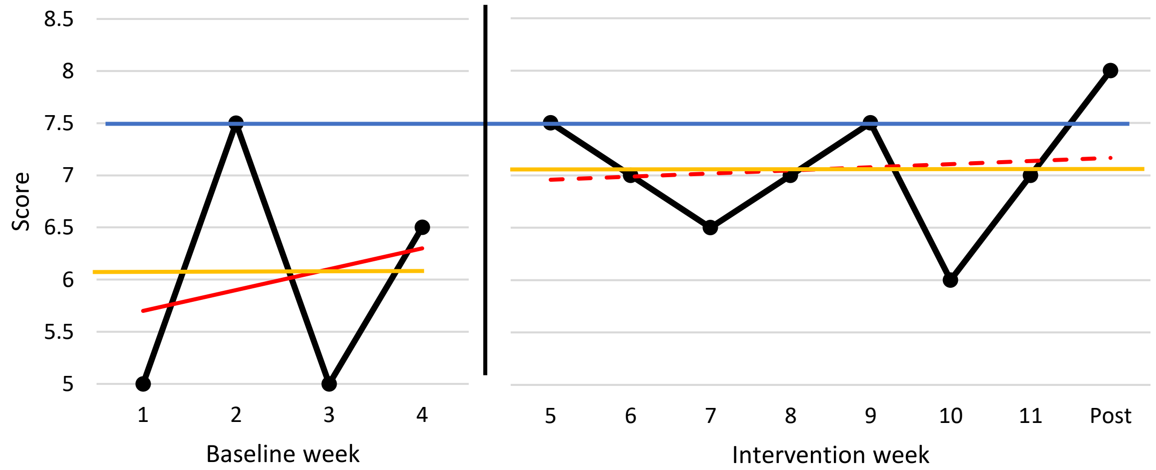

**ADL**	**Folding a blouse**
PQRS improvement	0.5 pts
Standard mean difference	0.87 pts
PND	12.50% (*p=0.54*)

Participant 15

ADL 1

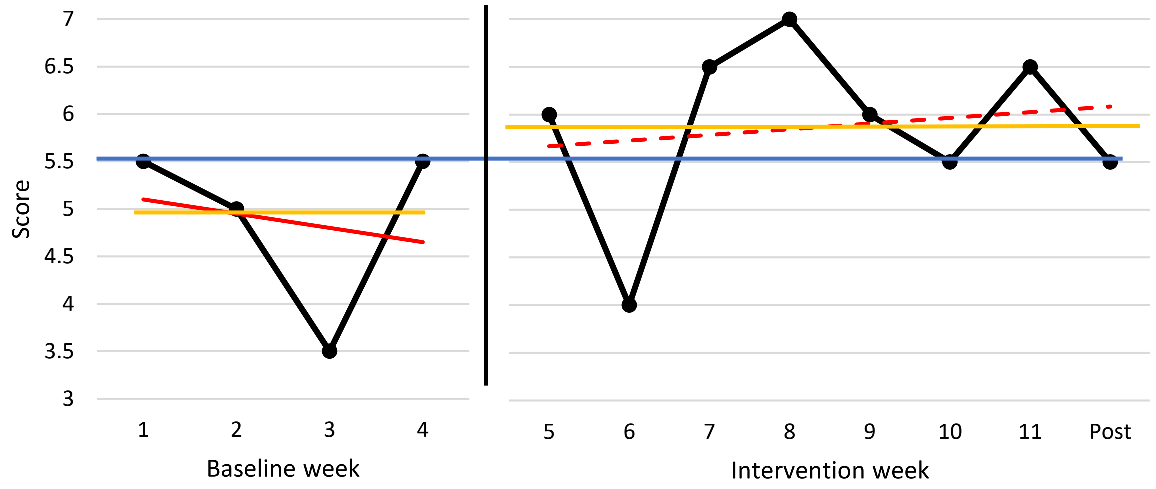

ADL	Putting on and buttoning up the school shirt
PQRS improvement	1.5 pts
Standard mean difference	1.06 pts
PND	50% (*p=0.11*)

ADL 2

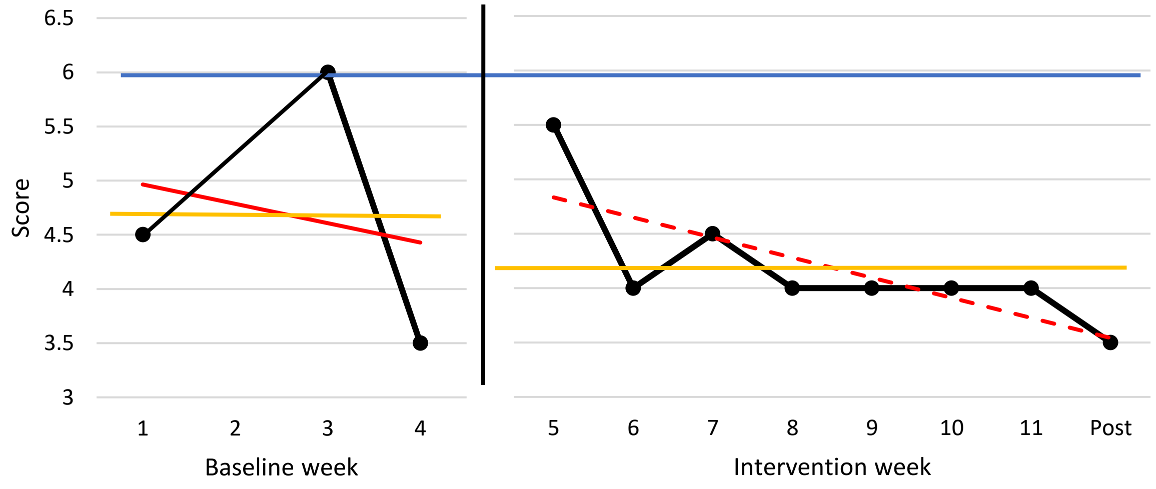

Missing data at baseline week 2.

ADL	Putting on shoes
PQRS improvement	0 pts
Standard mean difference	-0.38 pts
PND	0% (*p=0.62*)
