# Supplementary material for: Feasibility of a home-based home videogaming intervention with a family-centered approach for children with cerebral palsy: a randomized multiple baseline single-case experimental design
Source: J Neuroeng Rehabil. 2024 Sep 4;21:151. doi: 10.1186/s12984-024-01446-2 (PMC11373410; doi:10.1186/s12984-024-01446-2)

Appendix 5. Secondary outcomes descriptive statistics

Box and Blocks descriptive statistics

	N	Missing	Mean	Standard deviation
**Pre**	12	1	21.25	11.44
**Post**	12	1	23.08	9.99

Box and Blocks paired sample t-test (Student's t-test)

	t	df	p	Cohen's d	SE Cohen's d	95% CI for Cohen's d	
						Lower	Upper
**Pre-Post**	-1.229	11	0.25	0.36	0.14	-0.24	0.93

Example of CHEQ raw score data

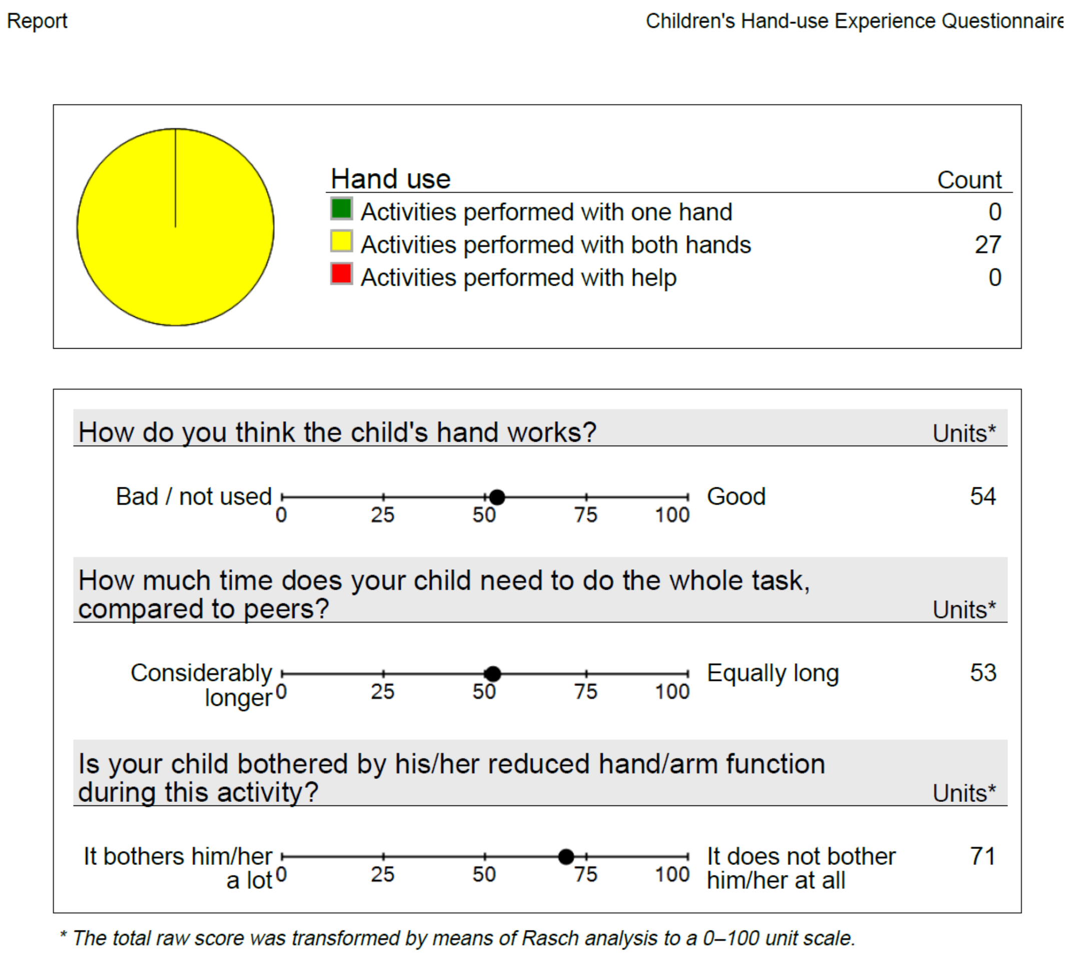

Supplement: Supplementary file 5 — Supplementary Material 5 [file 12984_2024_1446_MOESM5_ESM.pdf]
